# Elastic Behavior of Group 13–Phosphide Nanosheets and Nanotubes: A Numerical Study

**DOI:** 10.3390/nano16211398

**Published:** 2026-10-31

**Authors:** Nataliya A. Sakharova, André F. G. Pereira, Jorge M. Antunes

**Affiliations:** 1 UnIRE, Instituto Superior de Engenharia de Lisboa, Universidade Politécnica de Lisboa, Rua Conselheiro Emídio Navarro 1, 1959-007 Lisboa, Portugal; 2 Centre for Mechanical Engineering, Materials and Processes (CEMMPRE)—Advanced Production and Intelligent Systems, Associated Laboratory (ARISE), Department of Mechanical Engineering, University of Coimbra, Rua Luís Reis Santos, Pinhal de Marrocos, 3030-788 Coimbra, Portugal; andre.pereira@dem.uc.pt (A.F.G.P.); jorge.antunes@dem.uc.pt (J.M.A.); 3 Abrantes High School of Technology, Polytechnic Institute of Tomar, Quinta do Contador, Estrada da Serra, 2300-313 Tomar, Portugal

**Keywords:** group 13 elements, phosphide nanosheets, phosphide nanotubes, Young’s and shear moduli, modeling, numerical simulation

## Abstract

Group 13–phosphide nanosheets and nanotubes represent a family of nanostructures whose mechanical properties remain unevenly explored, particularly across the complete B–Al–Ga–In–Tl series. In this work, the elastic behavior of boron phosphide, aluminum phosphide, gallium phosphide, indium phosphide and thallium phosphide nanosheets and single-walled nanotubes is systematically investigated using nanoscale continuum modeling based on molecular structural mechanics and finite element analysis. The surface Young’s and shear moduli of the nanosheets are evaluated and their variation along the group 13–phosphide series is analyzed. The corresponding nanotubes are investigated considering zigzag, armchair and chiral configurations with diameters above 1.3 nm to assess the influence of geometry and chirality on their elastic response. The results reveal an overall decrease in the elastic properties along the B–Al–Ga–In–Tl series, although the trend is not uniform for all compounds and properties. The surface Young’s moduli of the nanotubes are found to be comparable to those of the corresponding nanosheets, whereas their surface shear moduli are consistently higher, despite the different deformation modes involved. Overall, this study provides a consistent assessment of the mechanical behavior of both 2D and 1D group 13–phosphide nanostructures within a unified modeling framework, while also providing reference data for compounds whose elastic properties remain scarcely reported.

## 1. Introduction

Two-dimensional (2D) materials have attracted considerable research interest due to their unique structural, electronic and optical properties and their potential for applications in nanoscale devices. Following the discovery of graphene, the search for graphene-like materials with a hexagonal atomic arrangement has been extended to a wide range of elemental and compound systems. Among them, group 13–phosphide compounds, formed by boron (B), aluminum (Al), gallium (Ga), indium (In) and thallium (Tl) in combination with phosphorus (P), constitute an interesting family of group 13–15 semiconductor materials. In their 2D form, boron phosphide (BP), aluminum phosphide (AlP), gallium phosphide (GaP), indium phosphide (InP) and thallium phosphide (TlP) can form hexagonal monolayers composed of alternating group 13 and phosphorus atoms [1,2,3].

The stability and selected properties of these graphene-like phosphide nanosheets (NSs) have been investigated predominantly through theoretical approaches, particularly density functional theory (DFT) calculations [1,2,3,4,5,6,7]. These studies have predicted stable or energetically favorable monolayer structures for BP, AlP, GaP and InP, while also revealing electronic and optical characteristics that make these materials potentially attractive for nanoscale applications (see, for example, [8,9,10,11,12]). In contrast, TlP, the last member of the phosphide series considered in the present study, remains comparatively unexplored. Nevertheless, the stability of 2D TlP monolayers has been predicted theoretically [3], and subsequent studies have reported high carrier mobility [13] and considered TlP as a component of ternary alloys with GaP [14].

Group 13–phosphide compounds are well-known semiconductor materials with applications in electronic and optoelectronic devices, while their two-dimensional counterparts may offer additional opportunities for nanoscale applications. In particular, hexagonal BP has been proposed for electronic and optical nanodevices [10,11,15], energy-storage applications such as batteries [16,17], and as a building block for complex heterostructures [18,19]. The predicted electronic and optical properties of other group 13–phosphide monolayers, including AlP, GaP, InP and TlP, further highlight the potential of this family of 2D materials for future nanoscale applications [13,20]. Despite these theoretical advances, the experimental realization of free-standing hexagonal group 13–phosphide monolayers remains challenging. For example, although BP films and layers have been experimentally grown on different substrates, a free-standing honeycomb BP nanosheet has not yet been successfully isolated [8,12]. Thus, theoretical and numerical investigations remain essential for understanding the structural and physical behavior and assessing the potential of this class of 2D materials.

An important extension of research on graphene-like 2D materials is the development of the corresponding one-dimensional (1D) tubular structures. Conceptually, a nanotube (NT) can be regarded as a nanosheet rolled into a cylindrical configuration, introducing curvature while preserving the basic hexagonal atomic network. This dimensional transition from 2D nanosheets to 1D nanotubes provides an opportunity to investigate how changes in geometry and curvature influence the resulting material properties. A systematic comparison between nanosheets and their corresponding nanotubes can therefore contribute to understanding the relationship between the elastic behavior of these two structural forms and to evaluating their suitability for different nanoscale applications.

The successful fabrication and extensive use of carbon nanotubes have stimulated the prediction and synthesis of non-carbon tubular nanostructures based on graphene-like hexagonal lattices. Group 13–phosphide compounds can form such structures, resulting in boron phosphide (BPNTs), aluminum phosphide (AlPNTs), gallium phosphide (GaPNTs), indium phosphide (InPNTs) and thallium phosphide (TlPNTs) nanotubes. To date, crystalline InPNTs and GaPNTs have been experimentally synthesized [21,22,23,24], whereas BPNTs and AlPNTs have been investigated primarily through theoretical approaches [25,26,27,28]. Previous theoretical studies of these phosphide nanotubes have focused predominantly on their structural, electronic and optical characteristics, motivated by their potential applications in nanoelectronic and optoelectronic devices (see, for example, [28,29,30]). In contrast, their elastic properties have received considerably less attention [31,32,33]. To the best of our knowledge, TlPNTs have not yet been reported, either theoretically or experimentally. However, the predicted stability of graphene-like TlP monolayers, together with the structural relationship between the nanosheet and nanotube forms considered for the other group 13 phosphides, provides a basis for exploring TlP in a tubular configuration. In this context, when considering TlPNTs, the present study extends the analysis to the complete series of group 13–phosphide nanostructures, from B to Tl, enabling their elastic behavior to be systematically investigated across the group.

Understanding the elastic behavior of both nanosheets and nanotubes is important for assessing their potential use as structural or functional components in nanoscale devices, where deformation and mechanical stability can directly affect their performance and reliability. In this context, Young’s and shear moduli provide relevant measures for characterizing the elastic response and for examining the transition between the 2D and 1D forms.

In the present study, the elastic behavior of graphene-like BP, AlP, GaP, InP and TlP nanosheets and the corresponding single-walled nanotubes (SWBPNTs, SWAlPNTs, SWGaPNTs, SWInPNTs and SWTlPNTs) is systematically investigated by numerical simulation based on the nanoscale continuum modeling/molecular structural mechanics (NCM/MSM) approach. This methodology allows the mechanical behavior of nanostructures to be modeled with relatively low computational cost, while remaining suitable for systems containing a large number of atoms. The surface Young’s and shear moduli of the phosphide nanosheets are evaluated and compared with those of their tubular counterparts. For the nanotubes, the analysis is restricted to structures with diameters larger than 1.3 nm. The study therefore provides a systematic assessment of the elastic response of group 13–phosphide nanostructures, considering their 2D nanosheet and 1D nanotube forms within a unified modeling framework.

## 2. Materials and Methods

### 2.1. Atomic Structure and Geometrical Characteristics of Group 13–Phosphide Nanosheets and Nanotubes

**Figure 1:**
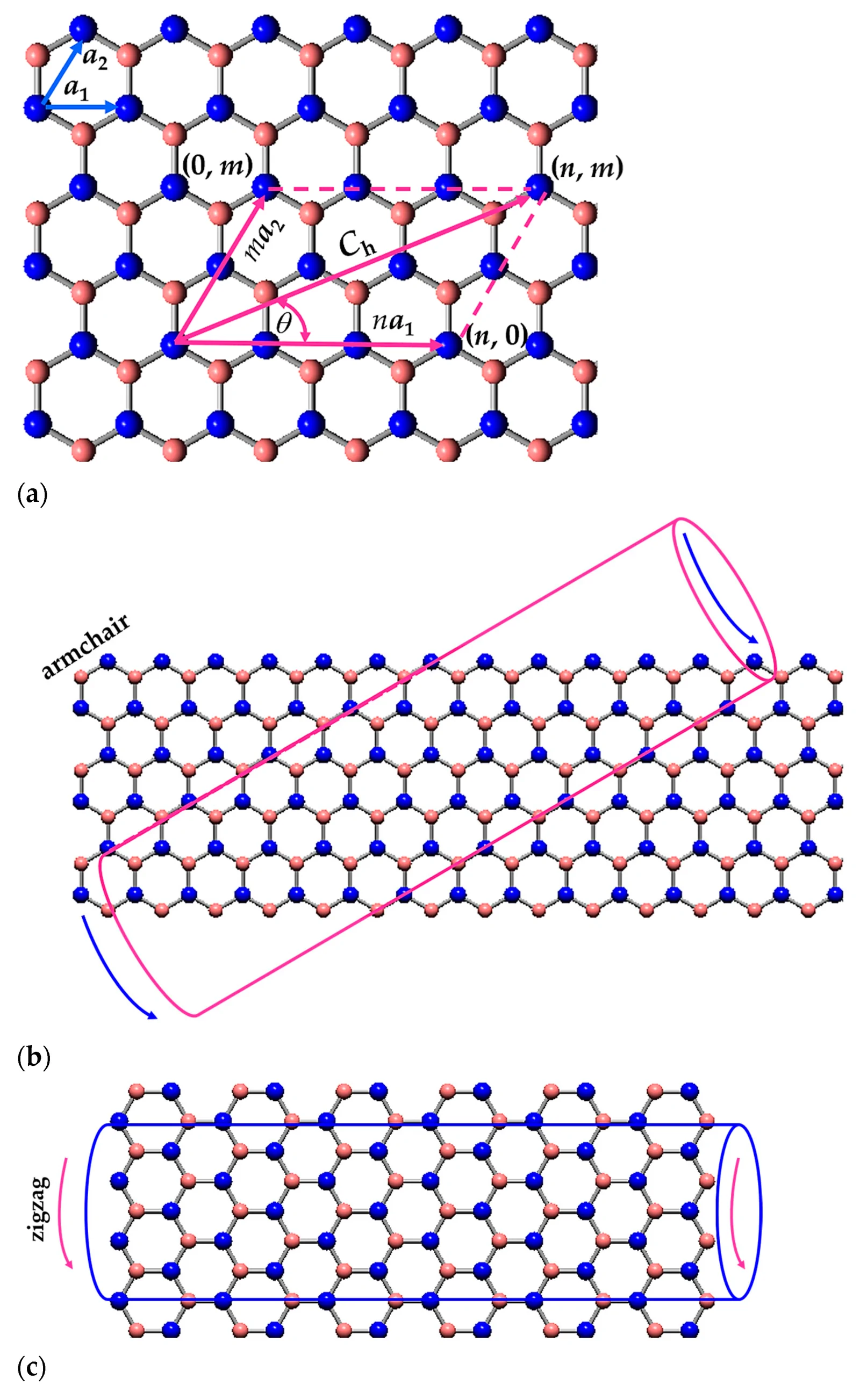
(**a**) Atomic structure of an AlP nanosheet showing the unit vectors $a_{1}$ and $a_{2}$, the chiral vector ***C*_h_**, chiral indices n and m, and the chiral angle *θ*. Schematic representation of the rolling direction leading to the nanotube configurations: (**b**) armchair and (**c**) zigzag. Al and P atoms are represented in shiny blue and pale orange, respectively.

The atomic structures considered in this study comprise hexagonal BP, AlP, GaP, InP and TlP nanosheets, in which phosphorus (P) atoms alternate with group 13 atoms—boron (B), aluminum (Al), gallium (Ga), indium (In) and thallium (Tl)—to form a honeycomb lattice. The atomic arrangement is illustrated in Figure 1a for the AlP nanosheet. Hereafter, the group 13 atom is denoted by A13. The geometry of the hexagonal lattice is described by the chiral vector, ***C*_h_**, and the chiral angle, *θ*, given by

(1) $$C_{h}=na_{1}+ma_{2}$$

(2) $$\;\theta =\sin^{-1}\frac{\sqrt{3}}{2}\frac{m}{\sqrt{n^{2}+\mathrm{nm}+m^{2}}},$$

where n and *m* are integer chiral indices; $a_{1}$ and $a_{2}$ denote the unit vectors of the diatomic hexagonal lattice. Their magnitude, $a=\left|a_{1}\right|=\left|a_{2}\right|$, is related to the equilibrium A13–P bond length, $a_{A13–P}$, calculated by *a* = $\sqrt{3}a_{A13–P}$.

A single-walled phosphide nanotube can be geometrically constructed by rolling the corresponding A13–P nanosheet into a cylindrical structure. The rolling geometry is determined by the chiral vector, ***C*_h_**, with the corresponding chiral angle, *θ*, ranging from 0° to 30°. The diameter of the resulting nanotube, $D_{n}$, is given by

(3) $$D_{n}=\frac{a_{A13–P}\sqrt{3\left(n^{2}+\mathrm{nm}+{\;m}^{2}\right)}}{\pi }.$$

Based on the chiral angle, *θ*, phosphide NTs can be classified into three symmetry groups: zigzag (*n*, 0), corresponding to *θ* = 0°; chiral (*n*, *m*), characterized by 0° < *θ* < 30° $\left(n\;\neq \;m;\;n,m\;\neq \;0\right)$; and armchair (*n*, *n*), for which *θ* = 30°. Zigzag and armchair NTs represent the two limiting configurations of the chiral angle range and are therefore called non-chiral nanotubes. The rolling schemes that lead to the armchair and zigzag configurations are illustrated in Figure 1b,c, respectively.

**Figure 2:**
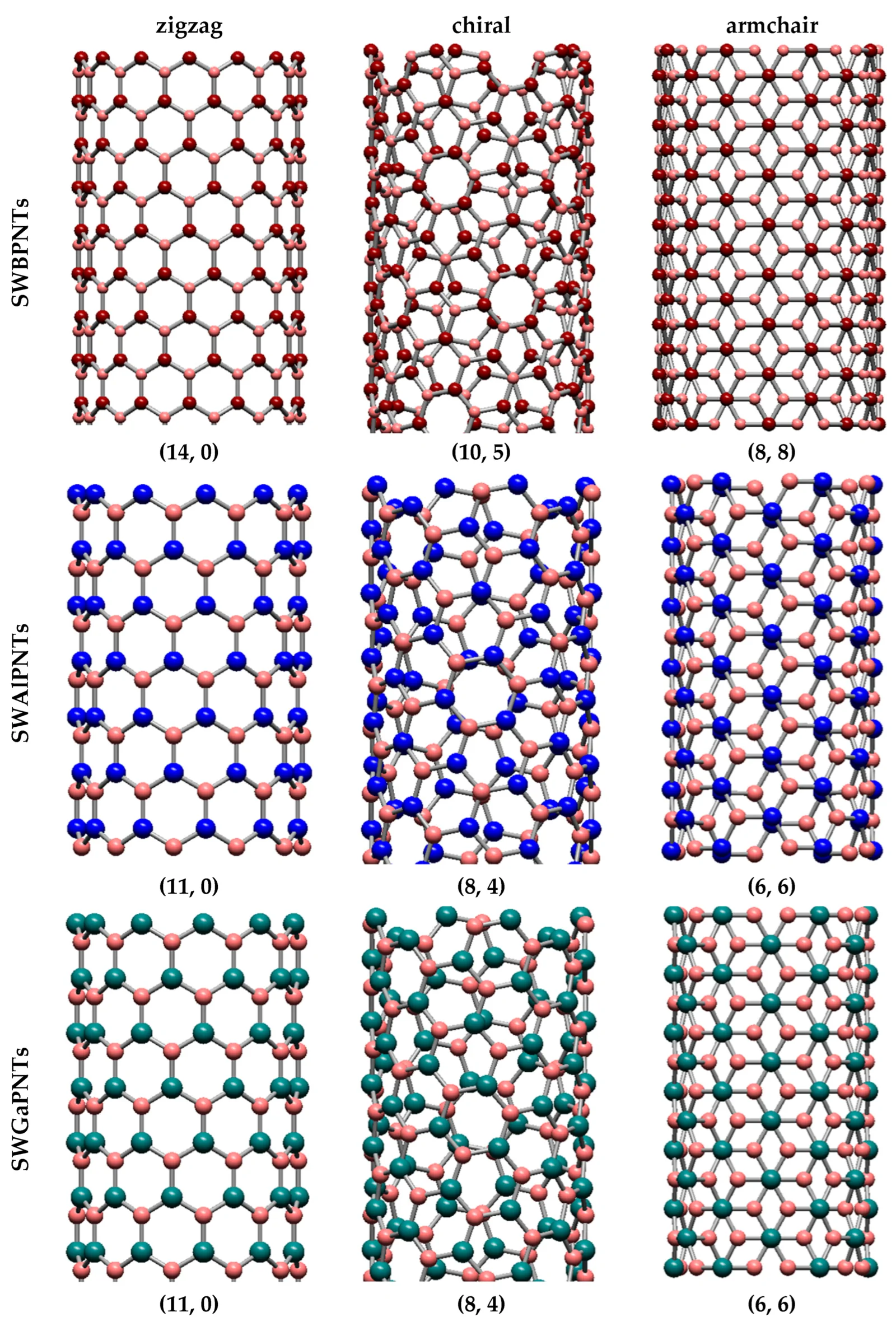

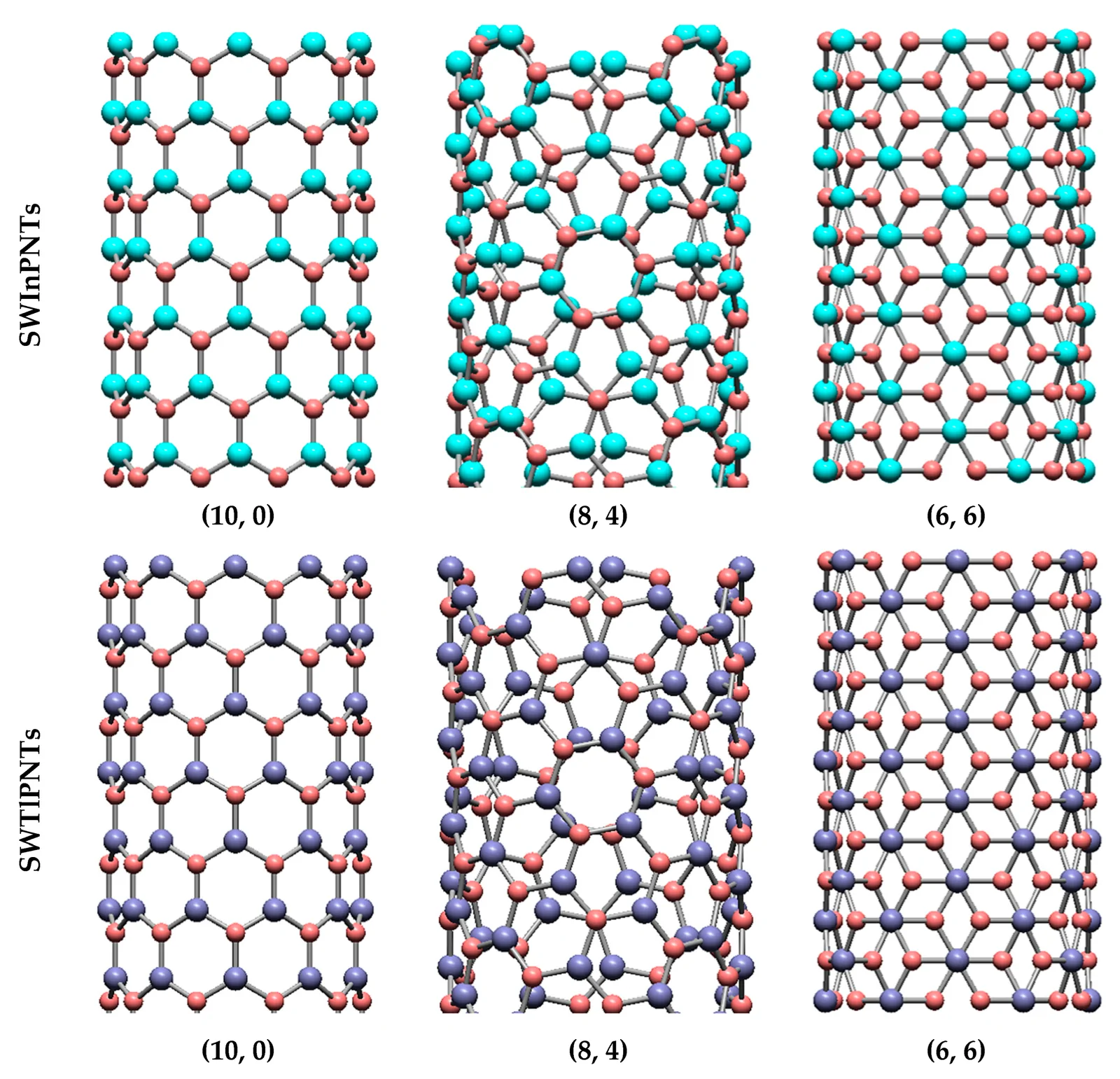
Representative structures of zigzag, chiral and armchair SWBPNTs, SWAlPNTs, SWGaPNTs, SWInPNTs and SWTlPNTs generated with Nanotube Modeler© software (version 1.8.0, ©JCrystalSoft, http://www.jcrystal.com). Phosphorus atoms are shown in pale orange, boron in bright red, aluminum in shiny blue, gallium in pale green, indium in turquoise and thallium in pale lilac.

Figure 2 shows representative examples of non-chiral (zigzag and armchair) and chiral SWBPNTs, SWAlPNTs, SWGaPNTs, SWInPNTs and SWTlPNTs. The chiral NTs belong to the most populated family considered in this study, with *θ* = 19.1°, while NTs with similar diameters were selected within each symmetry group. The three configurations were selected to examine the influence of chirality on the elastic response, covering the two limiting chiral angles and an intermediate configuration. Nanotube diameters above 1.3 nm were considered to reduce the influence of pronounced curvature effects associated with smaller nanotubes, thereby providing a more suitable basis for comparing their surface Young’s moduli with those of the corresponding planar nanosheets.

**Figure 3:**
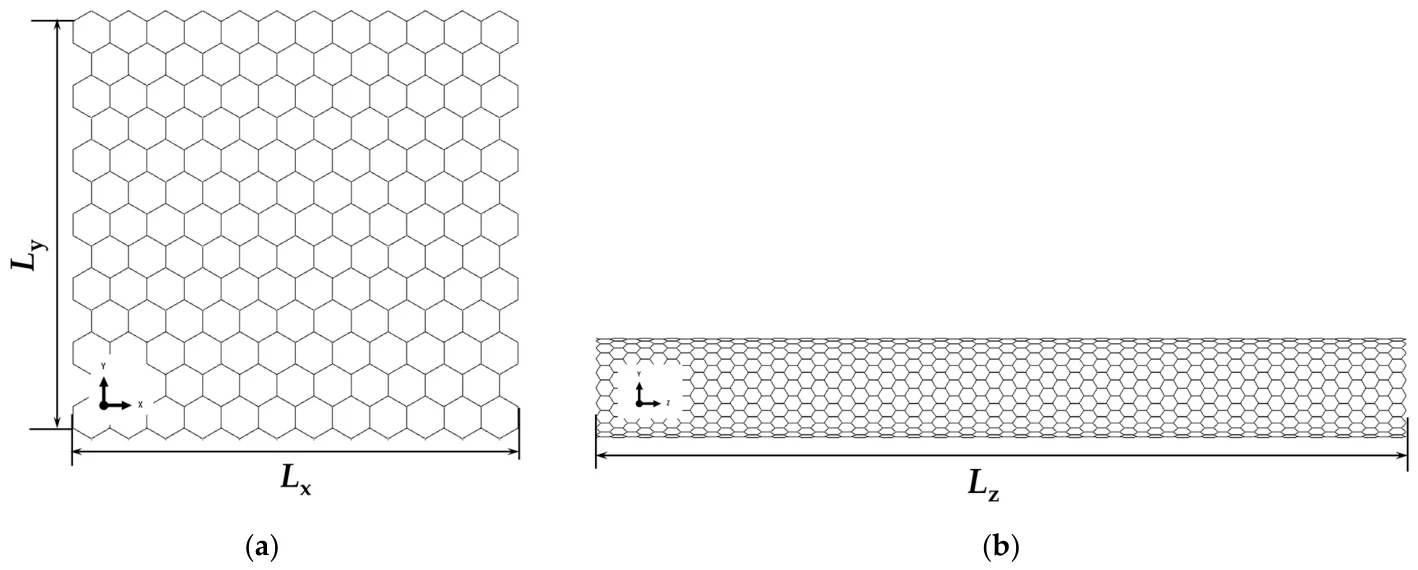
FE meshes representative of an AlP: (**a**) square single-layer nanosheet and (**b**) single-walled zigzag (20, 0) nanotube.

Based on the atomic structures described above, geometrical models of the phosphide NSs and NTs were prepared for subsequent numerical simulation. Following the generation of the atomic structures with Nanotube Modeler© software, the corresponding finite element (FE) meshes were generated, as illustrated for representative NS and NT models shown in Figure 3a,b, respectively.

The dimensions of the models were defined according to the coordinate systems shown in Figure 3. For the NSs, $L_{x}$ and $L_{y}$ denote the in-plane dimensions along the x- and y-directions, respectively, whereas $L_{z}$ represents the length of the NTs along their longitudinal axis. The main geometric characteristics of the NS and NT models considered in this study are summarized in Table 1 and Table 2, respectively.

### 2.2. Equivalent Beam Representation of Interatomic Bonds

The elastic behavior of group 13–phosphide nanostructures was modeled within the NCM/MSM framework. The combination of NCM/MSM with finite element analysis is particularly suitable for the present study, as it allows nanosheets and nanotubes with different geometries and atomic compositions to be analyzed within the same modeling framework. It also enables different loading conditions to be considered while retaining the connection between the molecular bond characteristics and the calculated mechanical response. This approach establishes a correspondence between the molecular nanostructure and its equivalent continuum structure, such that each diatomic bond is represented by an equivalent beam element [36]. Based on the energy equivalence between the molecular bond and the corresponding beam element, the bond stretching, *k*_r_, bond bending, *k*_θ_, and torsional resistance, *k*_τ_, force constants are related to the tensile, $E_{b}A_{b}$, bending, $E_{b}I_{b}$, and torsional, $G_{b}J_{b}$, rigidities of the beam, respectively [36],

(4) $$E_{b}A_{b}=lk_{r},\;E_{b}I_{b}=lk_{\theta },\;G_{b}J_{b}=lk_{\tau },$$

where $A_{b}\;=\;\pi d^{2}/4$, $I_{b}\;=\;\pi d^{4}/64$ and $J_{b}\;=\;\pi d^{4}/32$ are the cross-sectional area, moment of inertia, and polar moment of inertia of the circular beam, respectively; d is the equivalent beam diameter, and l is considered equal to the equilibrium bond length, $a_{A13–P}$.

Expressions (4) enable the input parameters required for the numerical simulation to be determined from the $k_{r}$, $k_{\theta }$, and $k_{\tau }$ force field constants. For the phosphide nanostructures considered in this study, values of the bond stretching, $k_{r}$, and bond bending, $k_{\theta }$, force constants are largely unavailable in the literature. Therefore, in the present work, $k_{r}$ and $k_{\theta }$ were determined using analytical expressions from molecular mechanics (MM) that relate these force constants to the surface Young’s modulus, $E_{s}$, and Poisson’s ratio, ν, of the corresponding monolayer. The values of $E_{s}$ and ν required for these calculations can be obtained either from DFT calculations or from experimental measurements. For the BP, GaP and InP 2D monolayers, $E_{s}$ and ν were taken from the study by Şahin et al. [1], who determined these properties using first-principles plane-wave calculations within the DFT framework. For the AlP 2D monolayer, the corresponding values reported by Singh et al. [37], based on DFT calculations employing the Tersoff–Brenner (TB) potential, were adopted. For the TlP 2D monolayer, $E_{s}$ and ν were taken from the work of Liu et al. [3], who performed first-principles calculations using the Vienna Ab initio Simulation Package (VASP), with the exchange-correlation energy described within the generalized gradient approximation (GGA) using the Perdew–Burke–Ernzerhof (PBE) parametrization.

The bond stretching and bond bending force constants were derived from the surface Young’s modulus, $E_{s}$, Poisson’s ratio, ν, and equilibrium bond length, $a_{A13–P}$, according to the following expressions [38]:

(5) $$k_{r}\;=\;\frac{3E_{s}}{\sqrt{3}\left(1\;-\;\nu \right)},$$

(6) $$k_{\theta }=\frac{E_{s}a_{A13–P}^{2}}{2\sqrt{3}\left(1+3\nu \right)}.$$

In the absence of compound-specific values, the torsional resistance force constant, $k_{\tau }$, was adopted from the DREIDING force field [39], which provides a consistent parameterization of the torsional contribution based on the hybridization of the bonded atoms.

The equilibrium bond length, $a_{A13–P}$, surface Young’s modulus, $E_{s}$, Poisson’s ratio, ν, and the corresponding force constants, $k_{r}$, *k*_θ_ and *k*_τ_, adopted for the group 13–phosphide nanostructures studied in this work are summarized in Table 3.

Based on these parameters, the geometrical and elastic properties of the equivalent beam elements were determined and subsequently used as input data for the FE simulations. The resulting values are presented in Table 4.

### 2.3. Finite Element Analysis and Assessment of Elastic Properties of Phosphide Nanosheets and Nanotubes

The finite element analysis (FEA) of the elastic behavior of group 13–phosphide nanostructures was carried out using the ABAQUS^®^ FE code (Abaqus 2020, Dassault SystèmesR, Providence, RI, USA). To obtain a format compatible with this software, the Program Database files generated by Nanotube Modeler© were converted using the in-house application *InterfaceNanostructures.NM* [34,35].

The elastic response of boron phosphide, aluminum phosphide, gallium phosphide, indium phosphide and thallium phosphide nanosheets (BPNSs, AlPNSs, GaPNSs, InPNSs and TlPNSs, respectively) was investigated under two tensile loading conditions, along with in-plane shear loading.

**Figure 4:**
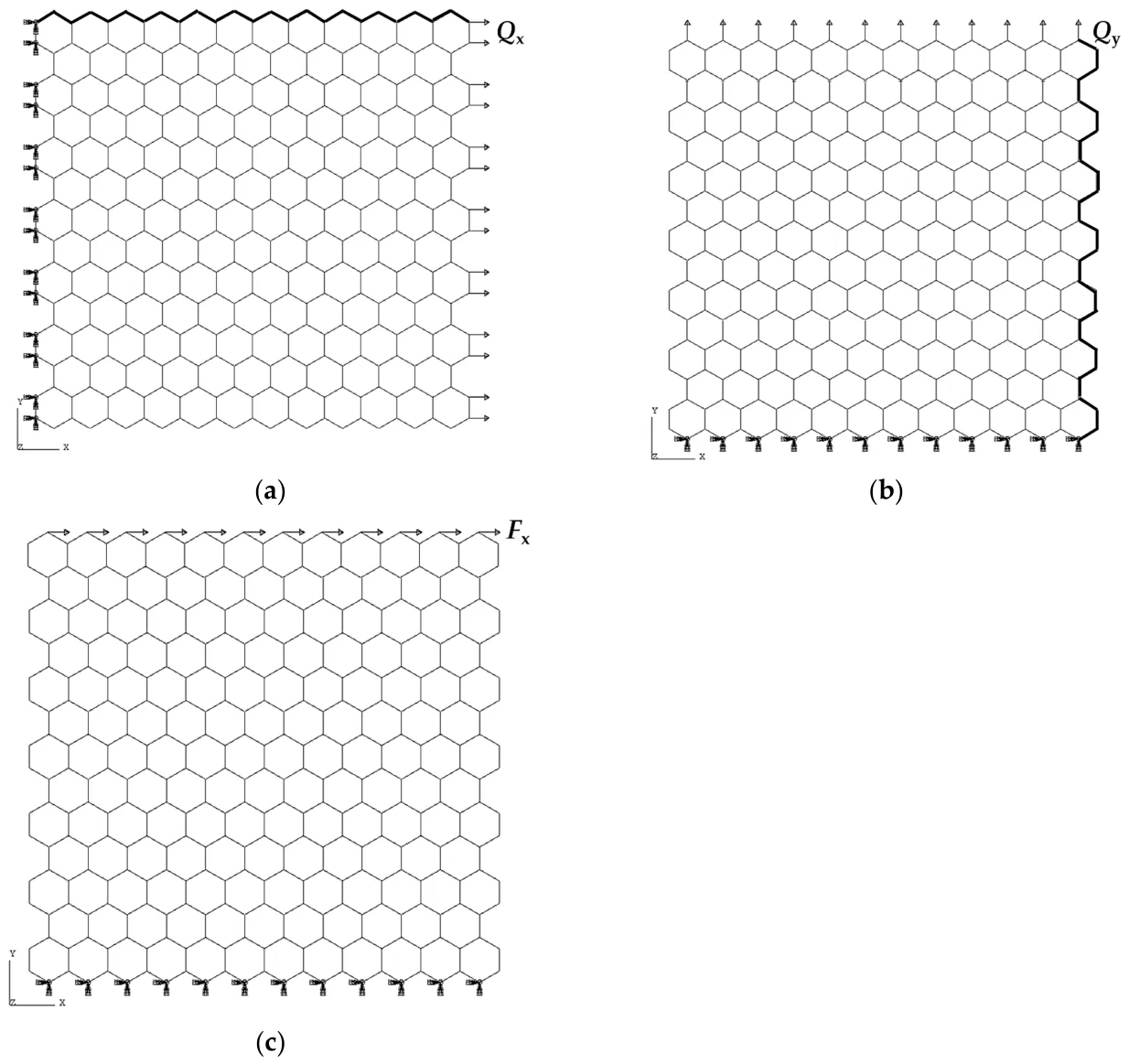
Schematic representation of the loading and boundary conditions applied to an AlPNS: (**a**) tensile loading in the x-direction (zigzag configuration); (**b**) tensile loading in the y-direction (armchair configuration); and (**c**) in-plane shear loading.

To simulate tension in the x-direction (zigzag configuration), a tensile force, $Q_{x}$, was applied to the nodes along the right edge of the NS, while the opposite edge was fixed (Figure 4a). The Young’s modulus in the x-direction, $E_{x}$, was calculated as [40]

(7) $$E_{x}=\frac{Q_{x}L_{x}}{s_{x}L_{y}t_{n}},$$

where $s_{x}$ is the NS displacement in the x-direction obtained from FEA, $L_{x}$ and $L_{y}$ are the NS side lengths (Figure 3a), and $t_{n}$ is the nanosheet thickness.

Similarly, tension in the y-direction (armchair configuration) was simulated by applying a tensile force, $Q_{y}$, to the nodes along the upper edge of the NS, while the lower edge remained fixed (Figure 4b). The Young’s modulus in the y-direction, $E_{y}$, was determined from [40]

(8) $$E_{y}=\frac{Q_{y}L_{y}}{s_{y}L_{x}t_{n}}$$

where $s_{y}$ is the NS displacement in the y-direction obtained from FEA.

For the in-plane shear test, a shear force, $F_{x}$, was applied to the nodes along the upper edge of the NS, while the lower edge was fixed (Figure 4c). The NS shear modulus, $G_{\mathrm{xy}}$, was then calculated as [40]

(9) $$G_{\mathrm{xy}}=\frac{F_{x}}{\gamma_{\mathrm{xy}}L_{x}t_{n}},\;\gamma_{\mathrm{xy}}=\tan\frac{u_{x}}{L_{y}},$$

where $u_{x}$ is the displacement in the x-direction obtained from FEA and measured in the central region of the nanosheet.

Since there is no well-defined thickness value, $t_{n}$, available for the group 13–phosphide NSs, their elastic response was characterized in terms of the surface Young’s moduli, $E_{\mathrm{sx}}$ and $E_{\mathrm{sy}}$, and surface shear modulus, $G_{\mathrm{sxy}}$, instead of the corresponding conventional moduli, $E_{x}$, $E_{y}$ and $G_{\mathrm{xy}}$. These surface elastic moduli are defined as the product of the respective elastic modulus and the nanosheet thickness. Accordingly, Equations (7)–(9) can be rewritten as

(10) $${E_{\mathrm{sx}}=E}_{x}t_{n}=\frac{Q_{x}L_{x}}{{s_{x}L}_{y}},$$

(11) $$E_{\mathrm{sy}}=E_{y}t_{n}=\frac{Q_{y}L_{y}}{s_{y}L_{x}},$$

(12) $$G_{\mathrm{sxy}}=G_{\mathrm{xy}}t_{n}=\frac{F_{x}}{\gamma_{\mathrm{xy}}L_{x}}.$$

**Figure 5:**
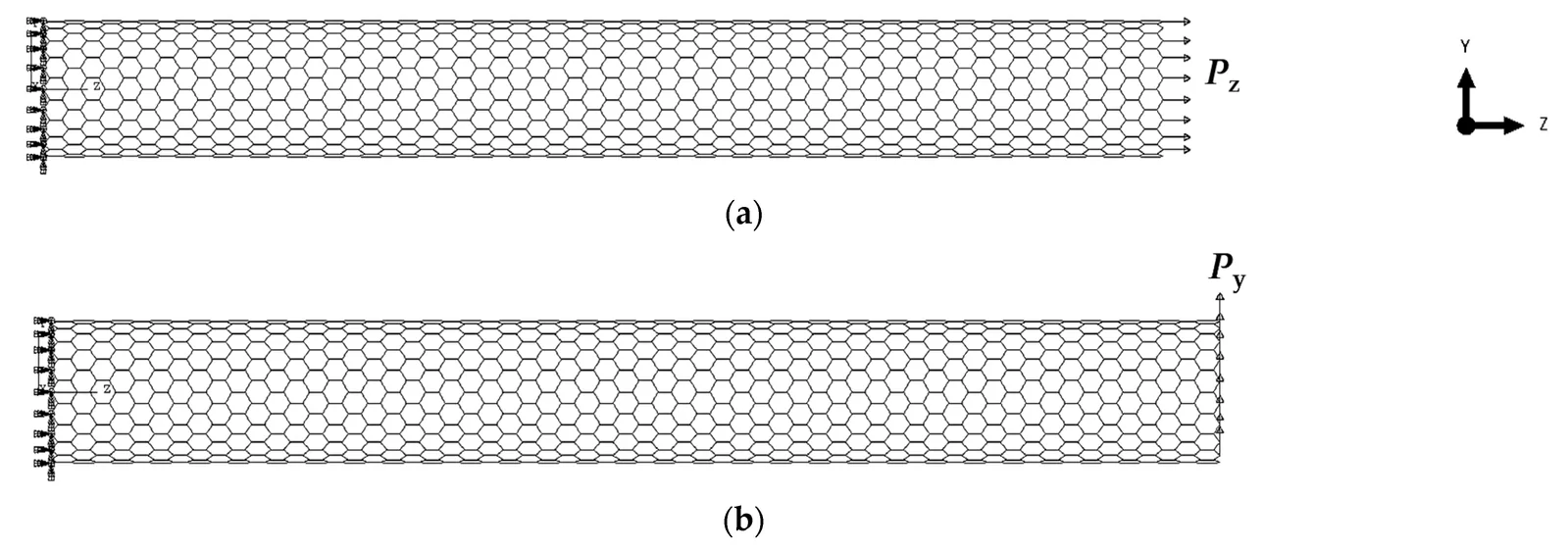

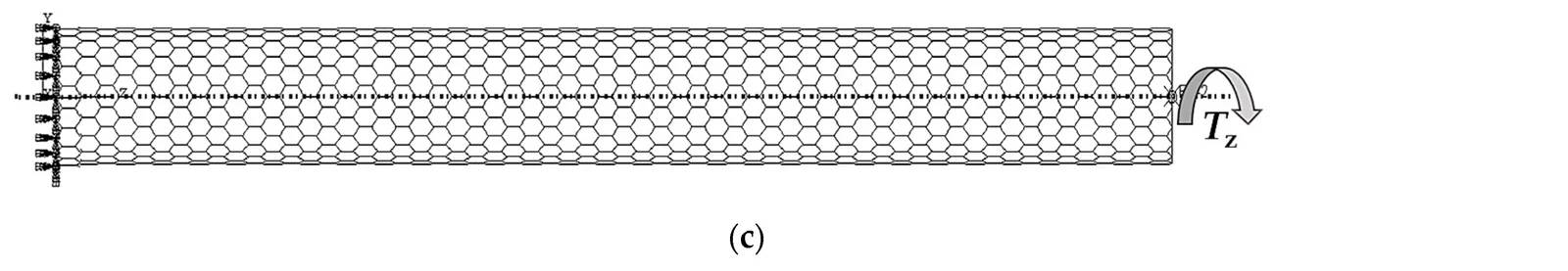
Loading and boundary conditions used for the numerical (**a**) tensile, (**b**) bending and (**c**) torsional tests, illustrated for a zigzag (20, 0) SWAlPNT.

The elastic responses of the single-walled BPNTs, AlPNTs, GaPNTs, InPNTs and TlPNTs were evaluated by numerical tensile, bending and torsional tests, whose loading and boundary conditions are illustrated in Figure 5. In all cases, one end of the NT was fully constrained, while the loading was applied to the opposite end. A longitudinal force, $P_{z}$, a transverse force, $P_{y}$, and a torque, $T_{z}$, were prescribed for the tensile, bending and torsional tests, respectively. For the torsional test, the radial displacement of the nodes at the loaded end was additionally restrained. The corresponding longitudinal displacement, $\delta_{z}$, transverse displacement, $\delta_{y}$, and twist angle, *φ*, were obtained directly from the FE simulations. These quantities, together with the applied loads, were subsequently used to calculate the tensile, *EA*, bending, *EI*, and torsional, *GJ*, rigidities of the NTs with length, $L_{z}$ (Figure 3b), according to

(13) $$EA=\frac{P_{z}L_{z}}{\delta_{z}},$$

(14) $$EI=\frac{P_{y}L_{z}^{3}}{{3\delta }_{y}},$$

(15) $$GJ=\frac{T_{z}L_{z}}{\varphi }.$$

The Young’s modulus, $E_{\mathrm{NT}}$, of the group 13–phosphide NTs can be determined by combining the tensile, *EA*, and bending, *EI*, rigidities obtained from Equations (13) and (14), respectively [35],

(16) $$E_{\mathrm{NT}}=\frac{EA}{\pi t_{n}\sqrt{8\left(\frac{EI}{EA}\right)–{\;t}_{n}^{2}}}$$

where $t_{n}$ denotes the nanotube wall thickness. The evaluation of the shear modulus, $G_{\mathrm{NT}}$, additionally involves the torsional rigidity, *GJ*, given by Equation (15), together with *EA* and *EI* [35],

(17) $$G_{\mathrm{NT}}=\frac{GJ}{2\pi t_{n}\left(\frac{EI}{EA}\right)\sqrt{8\left(\frac{EI}{EA}\right)–{\;t}_{n}^{2}}}.$$

Since the wall thickness of phosphide NTs cannot be unambiguously established, the elastic behavior of the nanotubes was characterized in terms of the surface Young’s and shear moduli, $E_{s}^{\mathrm{NT}}$ and $G_{s}^{\mathrm{NT}}$, respectively. Unlike conventional moduli, *E* and *G*, these surface quantities are independent of the assumed wall thickness and therefore provide a more suitable description of the elastic response of the phosphide NTs. Taking $E_{s}^{\mathrm{NT}}\;={\;E}_{\mathrm{NT}}\;t_{n}$ and $G_{s}^{\mathrm{NT}}\;=\;G_{\mathrm{NT}}{\;t}_{n}$, and neglecting the term $t_{n}^{2}$ in Equations (16) and (17), the surface Young’s and shear moduli can be expressed, respectively, as

(18) $$E_{s}^{\mathrm{NT}}={\;E}_{\mathrm{NT}}\;t_{n}=\frac{EA}{\pi \sqrt{8\left(\frac{EI}{EA}\right)}},$$

(19) $$G_{s}^{\mathrm{NT}}=G_{\mathrm{NT}}{\;t}_{n}=\frac{GJ}{2\pi \left(\frac{EI}{EA}\right)\sqrt{8\left(\frac{EI}{EA}\right)}}.$$

## 3. Results and Discussion

### 3.1. Elastic Properties of Group 13−Phosphide Nanosheets

#### 3.1.1. Surface Young’s and Shear Moduli of Group 13–Phosphide Nanosheets

**Figure 6:**
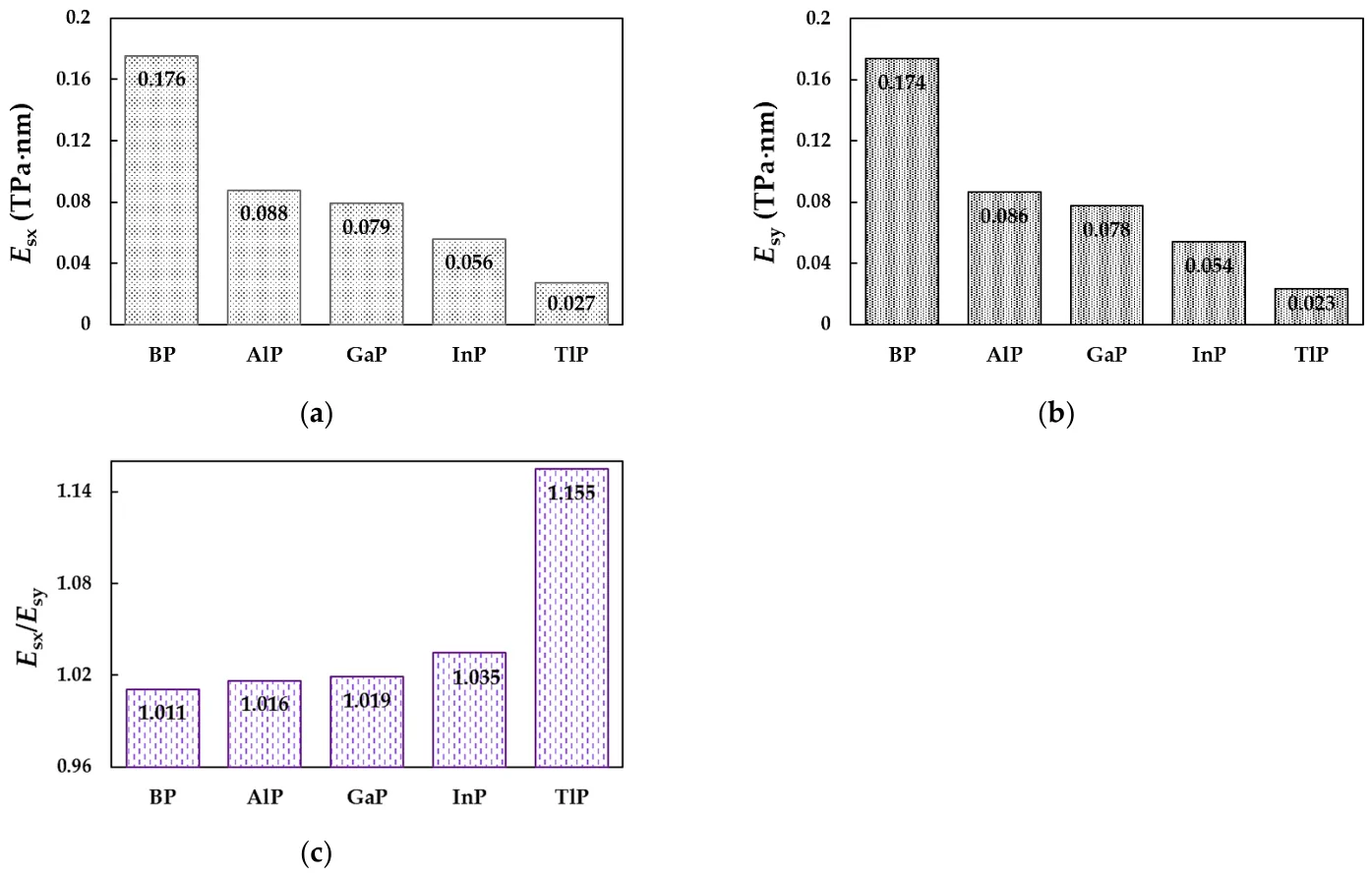
Variation of the surface Young’s modulus of group 13–phosphide nanosheets along the B–Al–Ga–In–Tl series: (**a**) $E_{\mathrm{sx}}$, corresponding to the zigzag direction; (**b**) $E_{\mathrm{sy}}$, corresponding to the armchair direction; and (**c**) $E_{\mathrm{sx}}/E_{\mathrm{sy}}$ ratio.

The surface Young’s moduli of the group 13–phosphide NSs in the x-direction (zigzag configuration, Figure 4a), $E_{\mathrm{sx}}$, and in the y-direction (armchair configuration, Figure 4b), $E_{\mathrm{sy}}$, were determined from Equations (10) and (11), respectively, based on the results of the numerical tensile tests. The calculated $E_{\mathrm{sx}}$ and $E_{\mathrm{sy}}$ values are presented in Figure 6a and Figure 6b, respectively, for BP, AlP, GaP, InP and TlP, following the order of the group 13 elements from B to Tl. Both surface Young’s moduli exhibit a progressive decrease along this sequence, with BPNS exhibiting the highest values and TlPNS the lowest. This trend indicates that the surface Young’s moduli decrease with the increasing atomic number of the group 13 elements. Figure 6c shows the ratio $E_{\mathrm{sx}}/E_{\mathrm{sy}}$ between the surface Young’s moduli in the zigzag and armchair directions. This ratio increases gradually from BPNS to InPNS and shows a more pronounced increase for TlPNS. Moreover, for all the phosphide NSs, $E_{\mathrm{sx}}$ > $E_{\mathrm{sy}}$, indicating some degree of in-plane elastic anisotropy.

The trends observed for the group 13–phosphide NSs cannot be directly related to the A13–P bond length. In our previous studies of the corresponding group 13–nitride nanosheets (boron nitride, aluminum nitride, gallium nitride, indium nitride and thallium nitride nanosheets—BNNSs, AlNNSs, GaNNSs, InNNSs and TlNNSs, respectively) [41,42], $E_{\mathrm{sx}}$ and $E_{\mathrm{sy}}$ exhibited decreasing trends with increasing bond length, whereas $E_{\mathrm{sx}}/E_{\mathrm{sy}}$ showed an increasing trend. However, establishing a similarly consistent relationship between these properties and bond length for the phosphide NSs is not straightforward. This difficulty arises primarily because the Al–P bond lengths reported in the literature are comparable to, or slightly greater than, the corresponding Ga–P bond length, complicating the identification of a systematic trend across the series.

In addition, the input parameters adopted for AlP in the present simulations were derived from material properties reported by Singh et al. [37], whereas those for the other phosphide compounds were obtained from different literature sources. To assess whether the choice of input data for AlP could influence the observed trends, three additional sets of parameters were considered. Specifically, the equilibrium bond length, $a_{\mathrm{Al}-P}\;=\;l$, surface Young’s modulus, $E_{s}$, and Poisson’s ratio, ν, reported by Liu et al. [3], Faraji et al. [5] and Kourra et al. [6], were used to recalculate the corresponding bond stretching and bond bending force constants, $k_{r}$ and $k_{\theta }$. All three studies employed the GGA with the PBE parametrization for the exchange-correlation energy. Liu et al. [3] and Faraji et al. [5] performed their calculations using VASP package, whereas Kourra et al. [6] employed the Quantum ESPRESSO package. Table 5 summarizes the material parameters, calculated force field constants, and resulting geometric and elastic properties of the equivalent beams for the three additional data sets. The corresponding parameters adopted in the present study, based on the data reported by Singh et al. [37], are also included for comparison.

Subsequently, FE simulations of the elastic response of the AlPNS listed in Table 1 were performed using the three additional sets of input parameters. The resulting surface Young’s moduli, $E_{\mathrm{sx}}$ and $E_{\mathrm{sy}}$, together with their ratio, $E_{\mathrm{sx}}/E_{\mathrm{sy}}$, are presented in Table 6.

As shown in Table 6, the surface Young’s moduli obtained for the zigzag and armchair directions are in reasonably close agreement for all sets of input parameters considered, and the $E_{\mathrm{sx}}/E_{\mathrm{sy}}$ ratio remains practically unchanged. The largest difference with respect to the $E_{\mathrm{sx}}$ and $E_{\mathrm{sy}}$ values adopted in the present study is approximately 8.8%, obtained using the parameters derived from the data reported by Kourra et al. [6]. When the parameters are based on the data of Liu et al. [3], the corresponding difference is about 3.3%. In contrast, the parameters derived from the values of l, $E_{s}$, and ν reported by Faraji et al. [5] lead to $E_{\mathrm{sx}}$ and $E_{\mathrm{sy}}$ values virtually identical to those obtained using the equivalent beam properties given in Table 4. The $E_{\mathrm{sx}}/E_{\mathrm{sy}}$ ratio varies only from 1.015 to 1.016 across all data sets considered. Therefore, although the choice of input parameters leads to some variation in the calculated surface Young’s moduli of AlPNS, it has a negligible effect on their relative values, and does not affect the trends observed in Figure 6.

**Figure 7:**
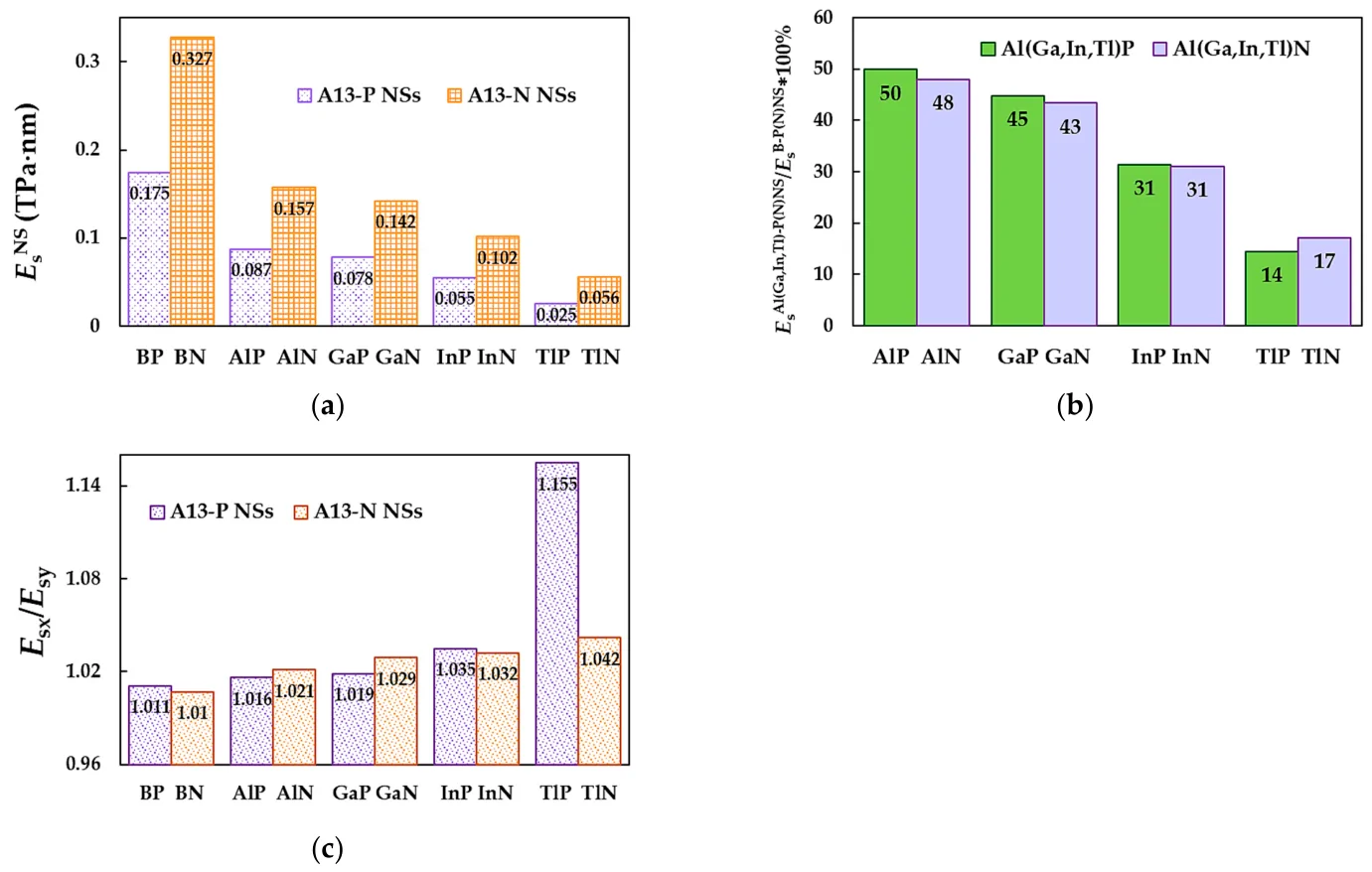
Comparison of the elastic response of group 13–phosphide and group 13–nitride nanosheets [41,42]: (**a**) average surface Young’s modulus, $E_{s}^{\mathrm{NS}}$; (**b**) average surface Young’s modulus normalized with respect to that of BPNS and BNNS for the phosphide and nitride series, respectively; and (**c**) $E_{\mathrm{sx}}/E_{\mathrm{sy}}$ ratio.

Following the representation adopted in Figure 6, Figure 7a compares the average surface Young’s moduli, $E_{s}^{\mathrm{NS}}\;=\;\left(E_{\mathrm{sx}}\;+{\;E}_{\mathrm{sy}}\right)/2$, of the group 13–phosphide NSs with those previously obtained for the corresponding group 13–nitride NSs. The comparison comprises compounds containing the same group 13 elements, namely, B, Al, Ga, In and Tl. The results for BNNSs were taken from the authors’ previous study [42], whereas those for AlNNSs, GaNNSs, InNNSs and TlNNSs were taken from [41]. In these studies, the $E_{\mathrm{sx}}$ and $E_{\mathrm{sy}}$ values reported were evaluated using the same modeling and calculation approaches as those employed in the present work.

As shown in Figure 7a, the average surface Young’s moduli of BPNSs, AlPNSs, GaPNSs and InPNSs are ≈1.8 times lower than those of their respective nitride counterparts. A somewhat larger difference is observed for TlPNS, whose $E_{s}^{\mathrm{NS}}$ is approximately 2.2 times lower than that of TlNNS. BPNS and BNNS exhibit the highest $E_{s}^{\mathrm{NS}}$ values within the phosphide and nitride series, respectively.

To further compare the evolution of the elastic response across these two series, Figure 7b shows the average surface Young’s moduli of AlPNSs, GaPNSs, InPNSs and TlPNSs normalized by that of BPNS, together with the corresponding values for AlNNSs, GaNNSs, InNNSs and TlNNSs normalized by that of BNNS. The normalized $E_{s}^{\mathrm{NS}}$ exhibit a remarkably similar reduction across the phosphide and nitride series from Al to In, whereas a larger difference between the two series appears for Tl. In particular, the normalized values decrease from approximately 50% for AlPNS and 48% for AlNNS to 31% for both InPNS and InNNS. For TlPNS and TlNNS, these values decrease further to approximately 14% and 17%, respectively. Thus, despite the considerable difference in the absolute surface Young’s moduli of the two compound families, their relative elastic response evolves in a remarkably similar manner across the group 13 elements.

This pronounced reduction in surface Young’s modulus along the group 13 series should be considered when assessing these phosphide nanosheets for applications in which mechanical stiffness is relevant. In particular, the substantially lower $E_{s}^{\mathrm{NS}}$ values obtained for InPNS and TlPNS indicate that their mechanical response may become an important design consideration when exploiting their electronic, optical or other functional properties in nanoscale devices.

Finally, Figure 7c compares the $E_{\mathrm{sx}}/E_{\mathrm{sy}}$ ratio across the phosphide and nitride series. Both series exhibit a gradual increase in this ratio along the group 13 sequence. However, while the increase remains relatively small for the nitride NSs, the phosphide series exhibits a more pronounced rise from InPNS to TlPNS, for which the ratio $E_{\mathrm{sx}}/E_{\mathrm{sy}}$ increases from 1.035 to 1.155, corresponding to an increase of approximately 11.6%.

**Figure 8:**
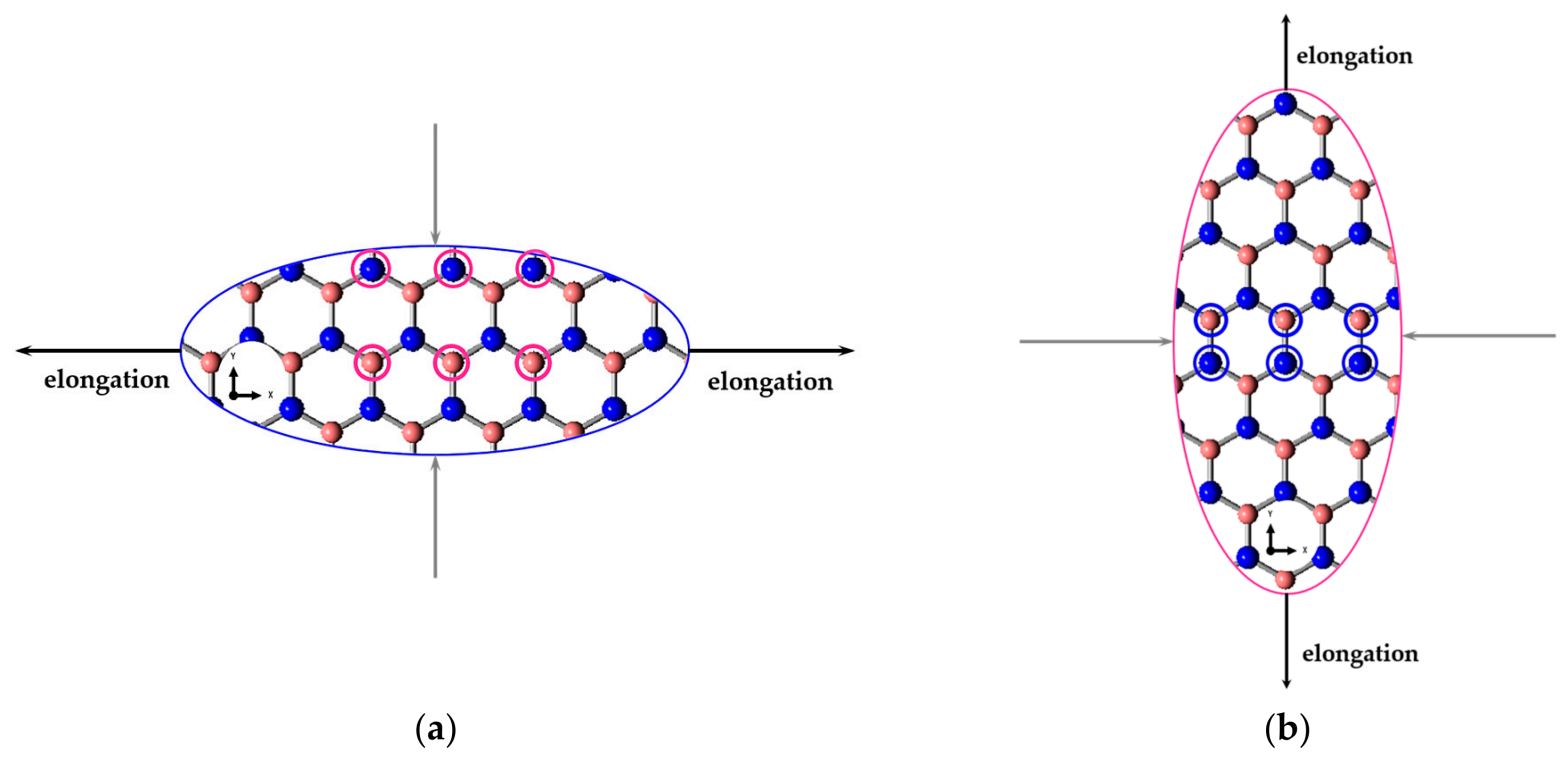
Schematic representation, for AlPNS, of the transverse contraction of the graphene-like phosphide NS lattice under tensile loading in the (**a**) zigzag and (**b**) armchair directions. Selected atoms illustrating the transverse contraction are highlighted. The grey arrows schematically indicate the transverse contraction associated with tensile loading.

As shown in Figure 6c and Figure 7c, $E_{\mathrm{sx}}>E_{\mathrm{sy}}$ for all the phosphide NSs, indicating in-plane elastic anisotropy. The difference between the two surface Young’s moduli is relatively small for BPNS, AlPNS, GaPNS and InPNS, but becomes considerably more pronounced for TlPNS. This directional dependence may be primarily associated with the geometrical arrangement of the hexagonal lattice and the distinct mechanisms by which transverse contraction is accommodated under tensile loading in the zigzag and armchair directions. As schematically illustrated in Figure 8 for AlPNS, the atomic arrangements involved in the transverse contraction of the nanosheet differ between the two loading configurations. Consequently, the lattice resistance to the associated transverse deformation may also vary, contributing to the observed difference between the surface Young’s moduli in the two directions. This anisotropy is particularly pronounced in TlPNS, as evidenced by its substantially higher $E_{\mathrm{sx}}/E_{\mathrm{sy}}$ ratio compared with those of the other phosphide NSs.

**Figure 9:**
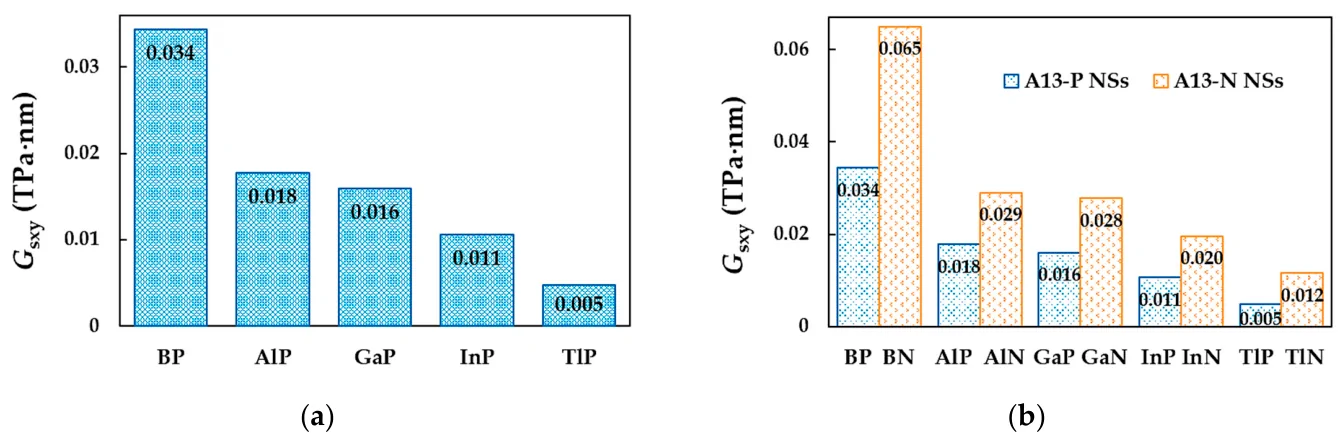
Surface shear modulus, $G_{\mathrm{sxy}}$, of group 13–phosphide nanosheets: (**a**) variation along the B–Al–Ga–In–Tl series; (**b**) comparison with the corresponding group 13–nitride nanosheets [41,42].

The surface shear modulus of the group 13–phosphide NSs, $G_{\mathrm{sxy}}$, was evaluated from Equation (12) using the results of the numerical in-plane shear tests (Figure 4c). The calculated $G_{\mathrm{sxy}}$ values are presented in Figure 9a for BP, AlP, GaP, InP and TlP, following the order of the group 13 elements from B to Tl. Similarly to the surface Young’s moduli, $E_{\mathrm{sx}}$ and $E_{\mathrm{sy}}$, the surface shear modulus decreases along this sequence, reaching its highest value for BPNS and its lowest for TlPNS. A pronounced reduction, of approximately 47%, occurs from BPNS to AlPNS, whereas AlPNS and GaPNS exhibit relatively similar $G_{\mathrm{sxy}}$ values. The surface shear modulus decreases further for InPNS and is reduced by more than half from InPNS to TlPNS. Overall, $G_{\mathrm{sxy}}$ exhibits a systematic decrease from B- to Tl-containing phosphide NSs, consistent with the general evolution already observed for $E_{\mathrm{sx}}$ and $E_{\mathrm{sy}}$.

It is worth noting that the $G_{\mathrm{sxy}}$ values calculated for AlPNS using the three additional sets of input parameters listed in Table 5, based on the data reported by Liu et al. [3], Faraji et al. [5] and Kourra et al. [6], are 0.018, 0.019 and 0.019 TPa·nm, respectively. These values are in close agreement with the value of 0.018 TPa·nm obtained using the input parameters adopted in the present study. The small differences between these results further suggest that the decrease in $G_{\mathrm{sxy}}$ across the phosphide series is insensitive to the set of input data considered for AlPNS.

Figure 9b compares the $G_{\mathrm{sxy}}$ values obtained in the present study for the group 13–phosphide NSs with those previously reported for the corresponding group 13–nitride NSs [41,42]. For BPNS, AlPNS, GaPNS and InPNS, the calculated values of $G_{\mathrm{sxy}}$ are, on average, about 1.8 times smaller than the corresponding values for the nitride series. This difference becomes more pronounced for the Tl-containing nanosheets, with the $G_{\mathrm{sxy}}$ of TlPNS being approximately 2.4 times smaller than that of TlNNS. Despite these differences in magnitude, the phosphide and nitride series exhibit a similar evolution of $G_{\mathrm{sxy}}$ from B to In, while a more pronounced separation between the two series occurs for Tl.

**Figure 10:**
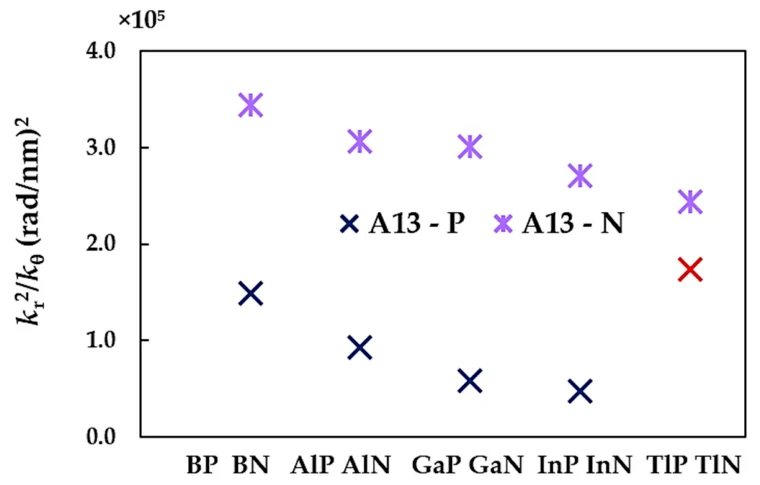
Variation of the $k_{r}^{2}/k_{\theta }$ ratio for group 13–phosphide and group 13–nitride nanosheets [41,42] along the B–Al–Ga–In–Tl series. The red symbol highlights the deviation of TlPNS from the trend observed for BPNS to InPNS.

The evolution of the elastic response of the group 13–phosphide NSs shown in Figure 6, Figure 7 and Figure 9 cannot be directly related to the A13–P bond length, indicating that bond length alone is insufficient to account for the variation in elastic properties across the phosphide series. Within the NCM/MSM framework adopted in the present study, the mechanical characteristics of the equivalent beam elements used in the FE simulations are derived from the molecular bond force constants (Table 4). Neither the bond-stretching force constant, $k_{r}$, nor the bond-bending force constant, $k_{\theta }$, considered individually reproduce the evolution observed for the calculated surface elastic moduli. However, these two force constants jointly determine the Young’s modulus of the equivalent beam, $E_{b}$, through a relationship involving the ratio $k_{r}^{2}/k_{\theta }$ ($E_{b}=\;k_{r}^{2}l/4\pi k_{\theta },$ where l is the bond length). This combined parameter was therefore considered to examine whether the evolution of the molecular bond characteristics within the adopted modeling framework is reflected in the elastic response of the NSs. Figure 10 shows the $k_{r}^{2}/k_{\theta }$ ratio for the phosphide and nitride NSs, arranged according to the group 13 element from B to Tl. For the phosphide series, $k_{r}^{2}/k_{\theta }$ decreases from BP to InP, while a generally decreasing evolution is also observed across the nitride series. This behavior is consistent with the overall decrease in the surface Young’s moduli, $E_{\mathrm{sx}}$ and $E_{\mathrm{sy}}$, and surface shear modulus, $G_{\mathrm{sxy}}$, observed in Figure 6a,b, Figure 7a,b and Figure 9a,b. In summary, it suggests that the combined contribution of bond stretching and bond bending provides a more meaningful descriptor of the evolution of the elastic properties than the individual force constants alone.

The value of $k_{r}^{2}/k_{\theta }$ obtained for TlPNS deviates markedly from the progression observed from BPNS to InPNS, as highlighted in Figure 10. Although the surface Young’s and shear moduli of TlPNS remain the lowest within the phosphide series, their evolution towards TlPNS cannot be fully explained by the $k_{r}^{2}/k_{\theta }$ ratio alone. Thus, this ratio provides a useful parameter for relating the evolution of the force field description to the general trends in the calculated elastic response, but the behavior of TlPNS indicates that this relationship should not be regarded as a direct or unique dependence. Notably, TlPNS also exhibits the most pronounced directional difference between $E_{\mathrm{sx}}$ and $E_{\mathrm{sy}}$, as evidenced by the considerably higher $E_{\mathrm{sx}}/E_{\mathrm{sy}}$ ratio. Within the adopted modeling framework, this distinctive behavior reflects the combined response of the equivalent beam elements representing the Tl–P bonds within the hexagonal lattice, although the present results do not allow it to be attributed to any individual beam parameter.

#### 3.1.2. Comparison with Literature Results

To place the present results in the context of previous theoretical predictions, the calculated elastic properties were compared with those reported in the literature for group 13–phosphide 2D structures, as summarized in Table 7. Most of the literature values considered for comparison were obtained from first-principles DFT calculations, predominantly using the Perdew–Burke–Ernzerhof (PBE) functional within the generalized gradient approximation (GGA), although different computational packages, including VASP [3,11,12,13,18,43], SIESTA [10] and Quantum ESPRESSO [15], were used. In contrast, Singh et al. [37] employed molecular dynamics (MD) simulations with a tight-binding (TB) potential to describe the interatomic interactions. The dimensions of the simulated nanosheets are generally not explicitly reported in the available studies, where the calculations are typically performed using a unit cell or supercell containing a limited number of atoms to represent the extended monolayer. Explicit nanosheet dimensions were only provided by Singh et al. [37].

Despite the differences between the modeling approaches, the present results are generally of the same order of magnitude as the available theoretical predictions, although the degree of numerical agreement varies across the reported values. As shown in Table 7, theoretical investigations of the elastic properties of these monolayers are relatively recent, with a substantial increase in the number of reported results in recent years. Within the group 13–phosphide series, BPNS has received considerably more attention than the other nanosheets. The closest agreement for BPNS is obtained with the surface Young’s modulus reported by Zhuang and Hennig [43], which differs from the present value by only about 2.0%. A reasonably close agreement was also obtained by Mohanta et al. [18], with a difference of approximately 12.6%. The results reported by Singh et al. [37], Gajjar et al. [11] and Vu et al. [15] are lower than the present value, while other available predictions show larger discrepancies [10,12]. For GaPNS and InPNS, the surface Young’s moduli values reported by Gajjar et al. [11] are approximately 30% lower than those obtained in the present study. For TlPNS, reasonably good agreement is observed between the present surface Young’s modulus in the armchair direction, $E_{\mathrm{sy}}$, and the value reported by Yuan et al. [13], with a difference of approximately 7.9%. It should be noted that Yuan et al. [13] studied ${\mathrm{TlP}}_{5}$ monolayers, which, although having a one-atom-thick structure, do not exhibit the honeycomb atomic arrangement considered in the present study.

Only a few studies have evaluated the surface Young’s moduli in both the zigzag and armchair directions, $E_{\mathrm{sx}}$ and $E_{\mathrm{sy}}$, respectively [11,18,37]. Among these, only Singh et al. [37] reported $E_{\mathrm{sx}}$ > $E_{\mathrm{sy}}$ for BPNS, in agreement with the directional dependence obtained in the present study. However, the corresponding $E_{\mathrm{sx}}/E_{\mathrm{sy}}$ ratio reported by these authors is slightly higher than the current value, indicating a somewhat more pronounced in-plane anisotropy. In contrast, Mohanta et al. [18] (for BPNS) and Gajjar et al. [11] (for BPNS, GaPNS and InPNS) reported surface Young’s moduli in the zigzag direction marginally lower than those in the armchair direction (Table 7). Nevertheless, the corresponding $E_{\mathrm{sx}}/E_{\mathrm{sy}}$ ratios are very close to 1.0, indicating a nearly isotropic in-plane elastic response.

With regard to the surface shear modulus of BPNS, most previous studies reported $G_{\mathrm{sxy}}$ values in the range 0.052–0.054 TPa·nm [3,10,11,15,37], or even higher [18], which are considerably greater than the value obtained in the present study. In contrast, the $G_{\mathrm{sxy}}$ value for BPNS calculated from the elastic constants reported by Galicia-Hernandez et al. [12] is only approximately 9.7% higher than the present result, showing reasonably good agreement. Literature data for the surface shear modulus are considerably scarcer for the other group 13–phosphide compounds. For AlPNS, the $G_{\mathrm{sxy}}$ values reported by Singh et al. [37] and Liu et al. [3] are approximately 8.1% and 10.5% higher, respectively, than that obtained in the present study.

**Figure 11:**
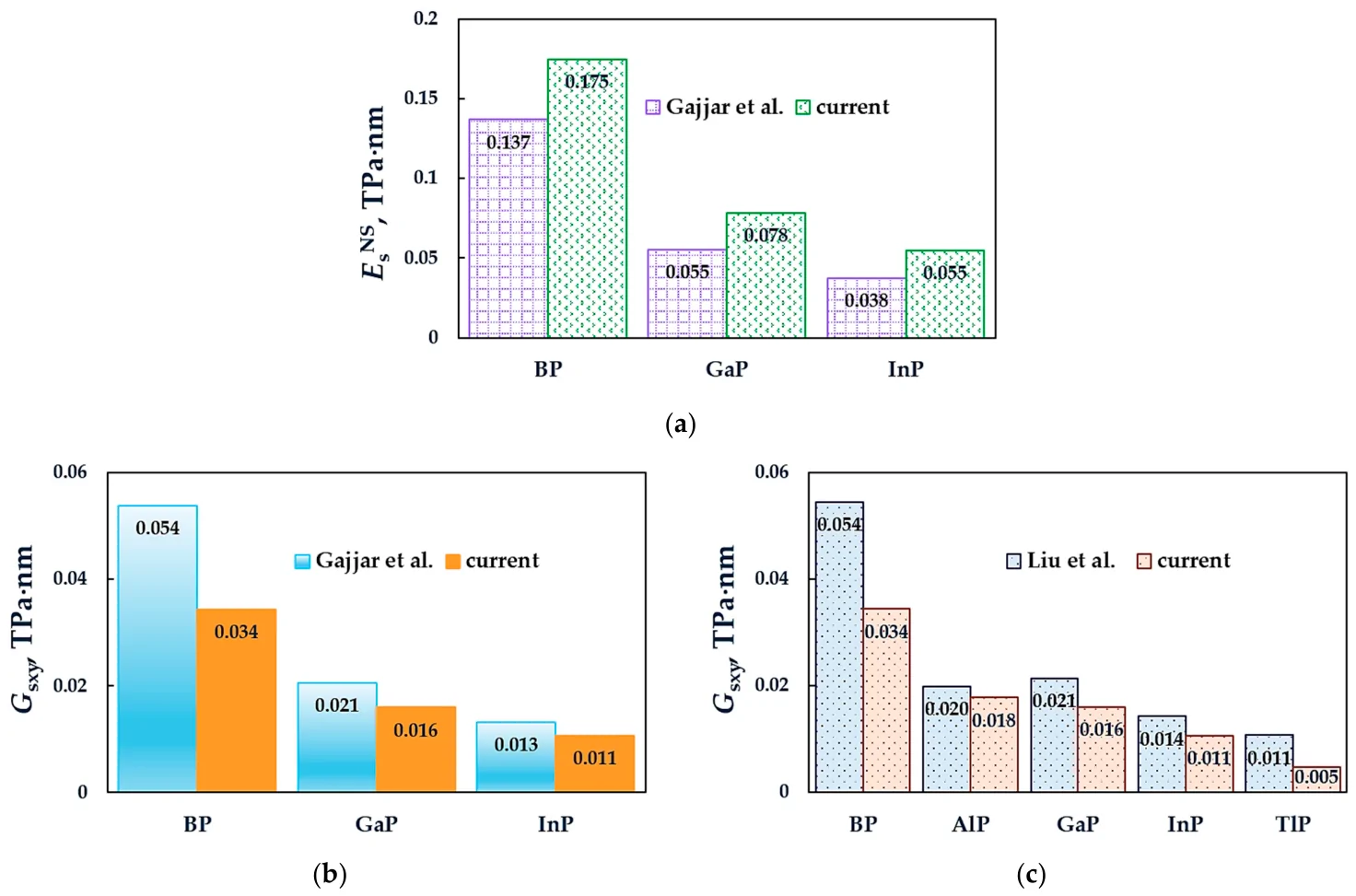
Comparison of the evolution of the elastic properties of the group 13–phosphide nanosheets obtained in the present study with literature results: (**a**) surface Young’s modulus, $E_{s}^{\mathrm{NS}}$, and (**b**) surface shear modulus, $G_{\mathrm{sxy}}$, for BP, GaP and InP nanosheets, using the data by Gajjar et al. [11]; (**c**) surface shear modulus, $G_{\mathrm{sxy}}$, along the B–Al–Ga–In–Tl series, using the data derived by Liu et al. [3].

To compare the evolution of the elastic properties along the group 13–phosphide series, the surface Young’s modulus values obtained in the present study are plotted in Figure 11a together with the available literature data, while the corresponding surface shear moduli are compared in Figure 11b,c. Despite the differences in the absolute values of the elastic moduli, the progressive decrease in both $E_{s}^{\mathrm{NS}}$ and $G_{\mathrm{sxy}}$ along the B–Ga–In sequence reported by Gajjar et al. [11] is qualitatively consistent with that observed in the present study (Figure 11a,b). Moreover, the relative reduction in the moduli values from one compound to another follows a broadly similar evolution.

The results of Liu et al. [3] provide a basis for comparing the surface shear modulus across the complete B–Al–Ga–In–Tl series (Figure 11c). Their results also show an overall decrease in $G_{\mathrm{sxy}}$ from BPNS to TlPNS, although the evolution is not strictly monotonic, as the value reported for AlPNS is slightly lower than that for GaPNS. Apart from this local inversion between AlPNS and GaPNS, the general evolution is qualitatively consistent with the systematic decrease in $G_{\mathrm{sxy}}$ obtained in the present study.

Thus, although the magnitude of the predicted elastic moduli depends on the modeling approach, the results available in the literature generally support their reduction along the group 13–phosphide series from B towards Tl. It should be noted that the current results were obtained using an FE model developed within the NCM/MSM framework, while the literature values considered for comparison were derived from atomistic methods, including first-principles calculations and MD simulations. By bringing together predictions obtained through fundamentally different modeling strategies, this comparison provides a broader benchmark for the elastic properties of these 2D structures.

To further contextualize the elastic behavior of group 13–phosphide nanosheets, the present results were also compared with the elastic properties reported for the corresponding three-dimensional (3D) materials. The studies selected for comparison were mainly theoretical, comprising DFT calculations [9,28,44,45,46,47,48,49,50,51,52,53,54,55], performed using different computational packages, including VASP [53], CASTEP [44,47,55], ABINIT [48,54], Quantum ESPRESSO [51] and CRYSTAL23 [28], as well as MD simulations [56,57]. Only in the case of InP, Pruessner et al. [58] assessed the Young’s modulus based on nanoindentation measurements. Regarding the crystal structures of the bulk phosphide compounds considered in these studies, different crystalline phases were investigated, including wurtzite (WZ) [9,47,53,54], zinc blende (ZB) [46,47,48,49,50,51,52,54], cubic [28,44], and hexagonal [55].

To allow comparison with the elastic moduli reported for the corresponding 3D compounds, an effective nanosheet thickness, $t_{n}$, was adopted, based on the study of Hess [59]. BPNS and AlPNS, with thickness values of 0.372 and 0.364 nm, respectively, were estimated from the corresponding van der Waals (vdW) diameters. In cases of GaPNS, InPNS and TlPNS, for which low-buckled monolayer configurations have been reported, the effective thickness values proposed by Hess [59] were adopted, which account for the buckling height in addition to the vdW diameter. The resulting $t_{n}$ values were 0.415, 0.429 and 0.442 nm for GaPNS, InPNS and TlPNS, respectively. Accordingly, the Young’s and shear moduli of the group 13–phosphide nanosheets were evaluated as

(20) $$E_{\mathrm{A13–P}}=\frac{E_{s}^{\mathrm{NS}}}{t_{n}},$$

(21) $$G_{\mathrm{A13–P}}=\frac{G_{s}^{\mathrm{NS}}}{t_{n}}.$$

For the adopted nanosheet thicknesses, the Young’s moduli calculated for the 2D group 13–phosphide structures are generally higher than those reported for their corresponding 3D compounds (Table 8). This trend is particularly pronounced for AlP, GaP and InP, for which the present values exceed all the literature results considered in the comparison. A similar tendency is observed for BP, although some reported 3D values are close to the present result. A different trend is found for the shear modulus. For BP, InP and TlP, the values reported for the corresponding 3D compounds exceed those obtained for the 2D monolayers. In the case of AlP, however, most of the literature values are close to the present result, indicating a comparable shear response. A similar agreement is observed for GaP in the results reported by Wang and Ye [54] and Ribeiro-Silva et al. [57], whereas the remaining literature values are higher than the present $G_{\mathrm{A13–P}}$ value. Overall, the comparison indicates that the corresponding 2D monolayers generally exhibit higher Young’s moduli than the 3D compounds, whereas a similar tendency is not observed for the shear modulus. Instead, the shear moduli calculated for the 2D structures are generally lower than those reported for the corresponding 3D values, with AlP representing the clearest exception among the compounds considered.

### 3.2. Elastic Properties of Group 13-Phosphide Nanotubes

#### 3.2.1. Rigidities of Single-Walled Group 13–Phosphide Nanotubes

**Figure 12:**
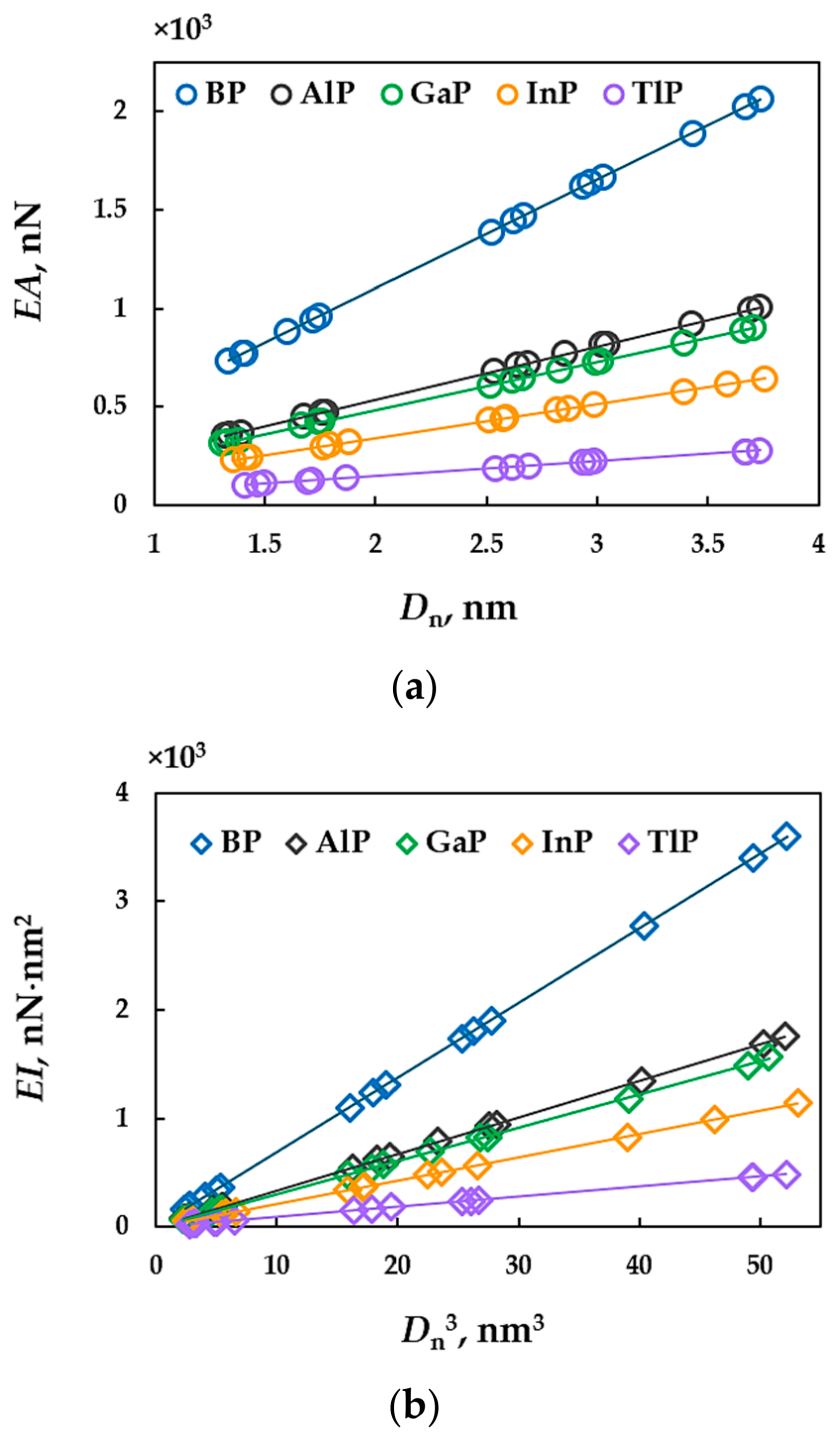

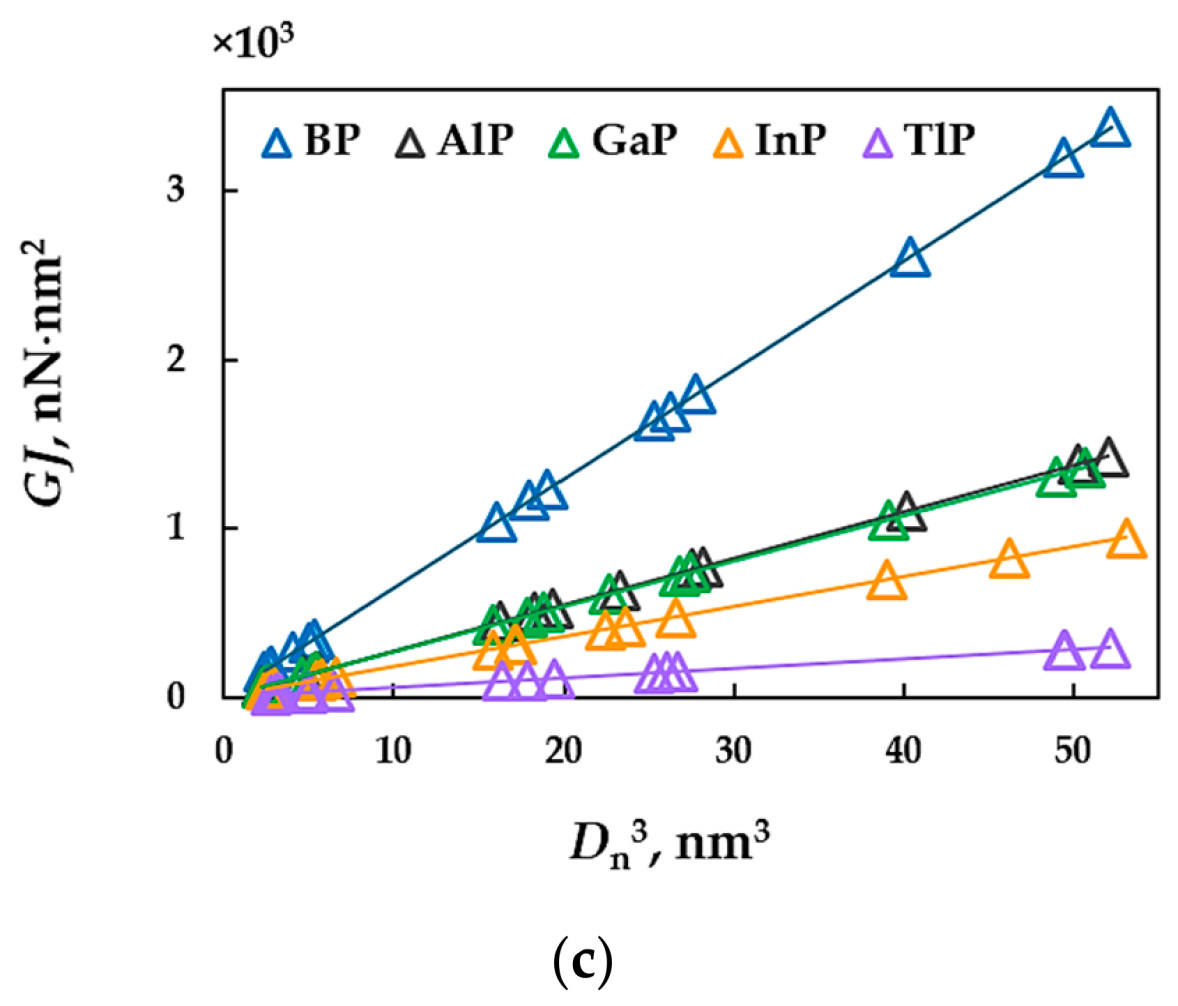
Linear fittings of the calculated rigidities for single-walled nanotubes of BP, AlP, GaP, InP and TlP listed in Table 2: (**a**) tensile rigidity, *EA*, as a function of nanotube diameter, $D_{n}$; (**b**) bending rigidity, *EI*, and (**c**) torsional rigidity, *GJ*, as functions of $D_{n}^{3}$.

The tensile, *EA*, bending, *EI*, and torsional, *GJ*, rigidities of the group 13–phosphide nanotubes were determined using Equations (13)–(15). Given the characteristic dependence of the mechanical rigidities of tubular structures on their diameter, the calculated *EA* values were analyzed as a function of nanotube diameter, $D_{n}$, whereas *EI* and *GJ* were examined as functions of $D_{n}^{3}$. As shown in Figure 12, all three rigidities exhibit approximately linear relationships, irrespective of nanotube chirality and the group 13–phosphide compound considered. The corresponding variations of *EA*, *EI*, and *GJ* rigidities with NT diameter, $D_{n}$, are presented in Figure S1 of the Supplementary Materials.

In addition to this common diameter dependence, clear differences in the magnitude of the rigidities are observed across the group 13–phosphide series. For a given nanotube diameter, *EA*, *EI* and *GJ* decrease markedly from SWBPNTs to SWAlPNTs. The values obtained for SWAlPNTs and SWGaPNTs are comparatively close, while a further progressive reduction is observed towards SWInPNTs and SWTlPNTs. The diameter dependence of the three rigidities can therefore be represented by the following analytical expressions, corresponding to the linear fittings shown in Figure 12:

(22) $$EA\;={\;\alpha }_{\mathrm{A13–P}}{\;D}_{n},$$

(23) $$EI={\;\beta }_{\mathrm{A13–P}}{\;D}_{n}^{3},$$

(24) $$GJ=\gamma_{\mathrm{A13–P}}{\;D}_{n}^{3},$$

Here, $\alpha_{\mathrm{A13–P}}$, $\beta_{\mathrm{A13–P}}$ and $\gamma_{\mathrm{A13–P}}$ are fitting parameters corresponding to the *EA*, *EI* and *GJ* rigidities, respectively.

The fitting parameters $\alpha_{\mathrm{A13–P}}$, $\beta_{\mathrm{A13–P}}$ and $\gamma_{\mathrm{A13–P}}$ were determined from the slopes of the linear regressions presented in Figure 12. All regressions yielded coefficients of determination, $R^{2}$, greater than 0.9999, confirming that the adopted diameter relationships provide an excellent representation of the numerical results. The resulting fitting parameters are listed in Table 9, together with the mean relative differences between the rigidities predicted by Equations (22)–(24) and those obtained directly from the FE simulations. These differences were evaluated by averaging the relative deviations calculated for the individual nanotubes of each compound.

The very small average relative differences reported in Table 9 further demonstrate the accuracy of the fitted analytical expressions. Across the five group 13–phosphide compounds, the average differences remain below 0.4% for *EA*, 0.9% for *EI*, and 0.6% for *GJ*. Thus, Equations (22)–(24), together with the corresponding fitting parameters, provide a simple and accurate means of estimating the tensile, bending, and torsional rigidities of the investigated nanotubes within the considered diameter range.

**Figure 13:**
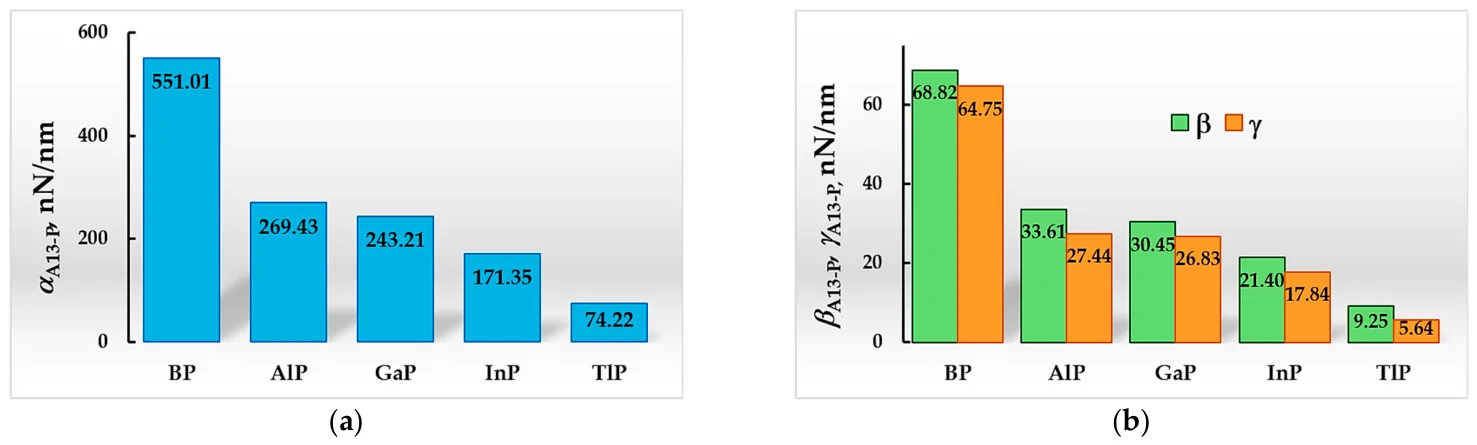

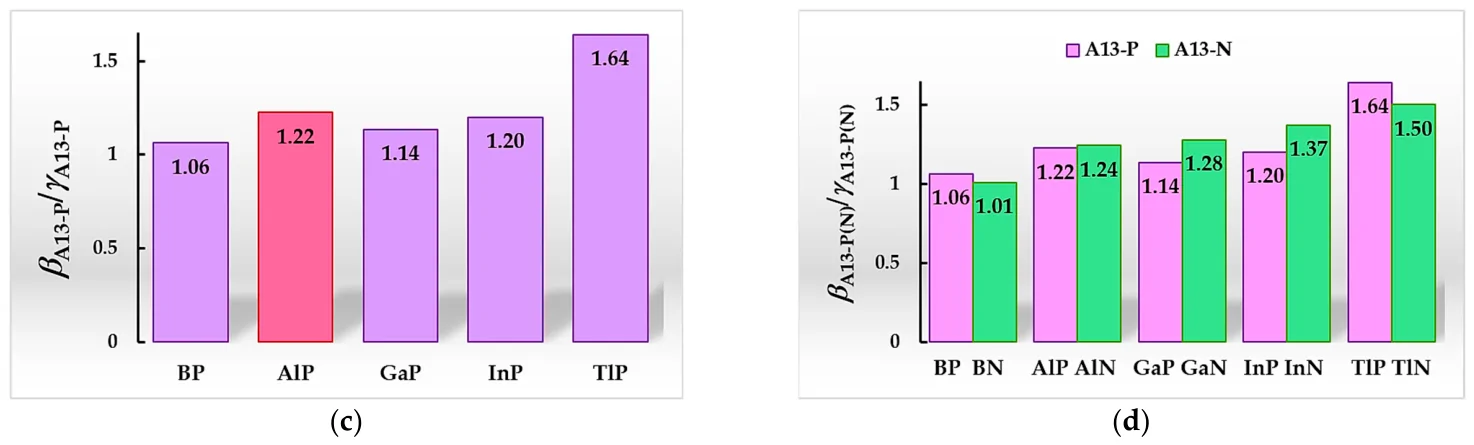
Evolution of the fitting parameters along the B–Al–Ga–In–Tl series: (**a**) $\alpha_{\mathrm{A13–P}}$; (**b**) $\beta_{\mathrm{A13–P}}$ and $\gamma_{\mathrm{A13–P}}$; (**c**) $\beta_{\mathrm{A13–P}}/\gamma_{\mathrm{A13–P}}$ ratio for the group 13–phosphide nanotubes; and (**d**) comparison of the β/γ ratios obtained for group 13–phosphide and group 13–nitride nanotubes [42,61]. The deep pink bar highlights the deviation of AlP from the general trend.

The fitting parameters provide a concise representation of how the magnitude of the nanotube rigidities varies across the group 13–phosphide series. As shown in Figure 13a,b, $\alpha_{\mathrm{A13–P}}$, $\beta_{\mathrm{A13–P}}$ and $\gamma_{\mathrm{A13–P}}$ follow a similar evolution from BPNTs to TlPNTs. A pronounced reduction occurs between BPNTs and AlPNTs, with all three parameters decreasing by approximately one half. The values obtained for AlPNTs and GaPNTs are comparatively close, followed by a progressive decrease through InPNTs to TlPNTs. This common evolution of the three fitting parameters reflects the systematic reduction in tensile, bending, and torsional rigidities across the phosphide series observed in Figure 12.

To further examine the relative evolution of the bending and torsional responses, the ratio between the corresponding fitting parameters, $\beta_{\mathrm{A13–P}}/\gamma_{\mathrm{A13–P}}$, is presented in Figure 13c. Since $\beta_{\mathrm{A13–P}}$ and $\gamma_{\mathrm{A13–P}}$ govern the diameter dependence of *EI* and *GJ*, respectively, their ratio provides a measure of the balance between the two rigidity contributions, independently of nanotube diameter. Overall, the ratio tends to increase along the BP–AlP–GaP–InP–TlP series, although the evolution is not monotonic: the relatively high value obtained for AlPNTs interrupts the progression from BPNTs to InPNTs. The most pronounced change occurs for TlPNTs, for which $\beta_{\mathrm{A13–P}}/\gamma_{\mathrm{A13–P}}$ reaches 1.64, compared with values ranging between 1.06 and 1.22 for the other phosphide nanotubes. This indicates that, for TlPNTs, the torsional rigidity decreases more strongly relative to the bending rigidity, leading to a great separation between the two deformation responses.

Interestingly, the distinctive behavior of TlPNTs is consistent with the evolution of the force field constants ratio $k_{r}^{2}/k_{\theta }$ previously discussed for the corresponding nanosheets (Figure 10). Although this ratio decreases progressively from BP to InP, TlP deviates markedly from this trend. Since the same force field constants are used to derive the elastic and geometrical properties of the equivalent beam elements in both the nanosheet and nanotube models, the behavior shown in Figure 10 is also relevant to the nanotube analysis. The simultaneous deviation of TlP from the general phosphide trend in both $k_{r}^{2}/k_{\theta }$ and $\beta_{\mathrm{A13–P}}/\gamma_{\mathrm{A13–P}}$ suggests a possible relationship between the particular combination of the Tl–P force field constants and the distinctive balance between the bending and torsional responses of TlPNTs.

A comparison with the corresponding fitting parameter ratios previously obtained for group 13–nitride nanotubes [42,61] provides further insight into this behavior. As shown in Figure 13d, the nitride series exhibits a progressive increase in $\beta_{A13-N}/\gamma_{A13-N}$ from boron nitride to thallium nitride nanotubes, BNNTs and TlNNTs, respectively, indicating a gradual change in the relative contributions of bending and torsional rigidities along the B–Al–Ga–In–Tl sequence. The phosphide nanotubes follow a less regular evolution. While the ratios for BPNTs, GaPNTs, and InPNTs remain below those of their nitride counterparts, with AlPNTs showing a very similar value to aluminum nitride nanotubes (AlNNTs), the relationship is reversed for the Tl-based compounds: TlPNTs exhibit a ratio of 1.64, exceeding the value of 1.50 obtained for TlNNTs. This distinctive behavior of TlPNTs is consistent with the deviation of TlP from the general phosphide evolution of $k_{r}^{2}/k_{\theta }$ observed in Figure 10. The comparatively smooth evolution observed for the nitride series contrasts with the departures exhibited by TlP in both $k_{r}^{2}/k_{\theta }$ and $\beta_{A13-N}/\gamma_{A13-N}$. This parallel behavior further supports the possible link between the force field constants and the relative bending and torsional responses suggested above, without implying a direct correspondence between the two ratios.

In addition, the sensitivity of the relative bending and torsional response to the input parameters listed in Table 5 was examined for an individual armchair SWAlPNT with chiral indices (17,17) and diameter $D_{n}\;=\;3.734$ nm. The EI/GJ ratio was found to be 1.03, 1.10, 1.23, and 1.28 when the input data from Faraji et al. [5], Liu et al. [3], Singh et al. [37] (adopted in the present study), and Kourra et al. [6], respectively, were employed. Although EI remains higher than GJ for all four parameter sets, the extent of the difference between the two rigidities varies considerably with the elastic and geometrical properties assigned to the equivalent beam elements. The results therefore show that the relative bending and torsional response predicted for the considered AlPNT is sensitive to the input parameters adopted in the FE model within the NCM/MSM approach.

#### 3.2.2. Elastic Moduli of Single-Walled Group 13–Phosphide Nanotubes

After analyzing the tensile and bending rigidities in Section 3.2.1, their relationship with the elastic response of the nanotubes was examined through the surface Young’s modulus, $E_{s}^{\mathrm{NT}}$. This modulus was evaluated using Equation (18), which relates $E_{s}^{\mathrm{NT}}$ to the *EA* and *EI* rigidities obtained from the FE simulations. As shown above, these rigidities can be accurately represented by the analytical relations given by Equations (22) and (23). Substituting these relations into Equation (18) eliminates the explicit dependence on nanotube diameter, and leads to the following expression:

(25) $$E_{s}^{\mathrm{NT}}\;=\frac{{\;\alpha }_{\mathrm{A13–P}}}{\pi \sqrt{8\left(\frac{{\;\beta }_{\mathrm{A13–P}}}{{\;\alpha }_{\mathrm{A13–P}}}\right)}},$$

Here, $\alpha_{\mathrm{A13–P}}$ and $\beta_{\mathrm{A13–P}}$ are the fitting parameters associated with tensile and bending rigidities, respectively, as reported in Table 9.

**Figure 14:**
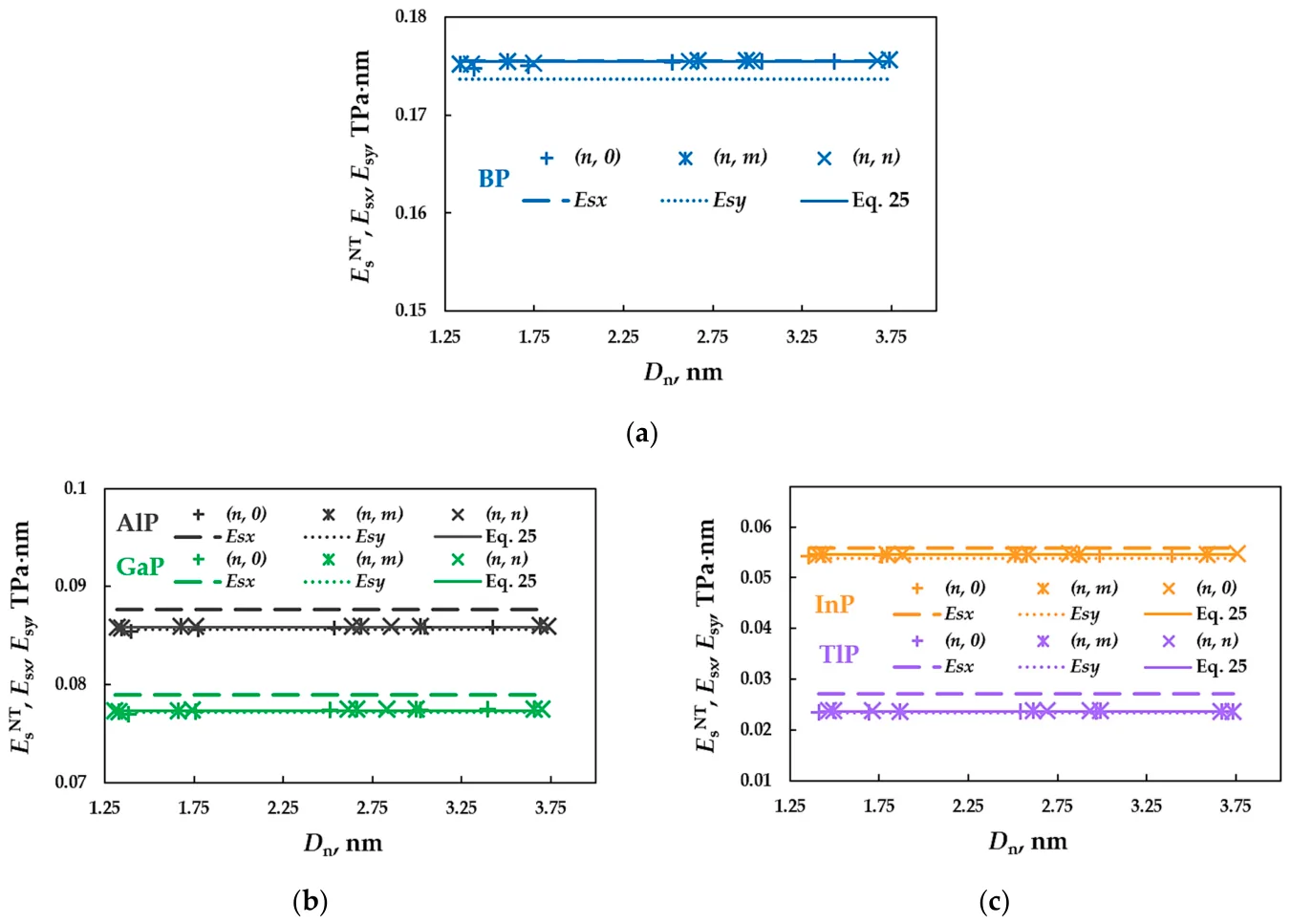
Surface Young’s modulus, $E_{s}^{\mathrm{NT}}$, as a function of nanotube diameter, $D_{n}$, for (**a**) SWBPNTs, (**b**) SWAlPNTs and SWGaPNTs, and (**c**) SWInPNTs and SWTlPNTs. The values predicted by Equation (25) and the surface Young’s moduli in the zigzag and armchair directions, $E_{\mathrm{sx}}$ and $E_{\mathrm{sy}}$, respectively, of the corresponding nanosheets are also shown.

Figure 14a–c presents the surface Young’s moduli, $E_{s}^{\mathrm{NT}}$, obtained directly from the FE rigidities through Equation (18) for all the nanotube configurations considered. The values predicted using Equation (25) are included for comparison, together with the surface Young’s moduli in the zigzag and armchair directions, $E_{\mathrm{sx}}$ and $E_{\mathrm{sy}}$, respectively, of the corresponding nanosheets. For each phosphide compound, the $E_{s}^{\mathrm{NT}}$ values remain nearly unchanged throughout the investigated diameter range, with no appreciable dependence on nanotube chirality. This behavior confirms that, for nanotubes with $D_{n}>1.3$ nm, the surface Young’s modulus is essentially independent of nanotube geometry within the range considered. Moreover, the values predicted by Equation (25) closely reproduce those calculated directly from the FE results. The mean relative differences are approximately 0.10% for BPNTs and AlPNTs, 0.20% for GaPNTs and InPNTs, and 0.30% for TlPNTs. Thus, the fitting parameters obtained from the tensile and bending rigidities provide, through Equation (25), an accurate estimate of $E_{s}^{\mathrm{NT}}$, without requiring the FE evaluation of individual nanotube configurations.

Comparison with the corresponding nanosheets reveals different relationship between the 1D and 2D surface Young’s moduli across the phosphide series. For BP, $E_{s}^{\mathrm{NT}}$ nearly coincides with the nanosheet modulus in the zigzag direction, $E_{\mathrm{sx}}$, whereas for AlP, GaP, InP, and TlP it lies considerably closer to the corresponding armchair value, $E_{\mathrm{sy}}$ (Figure 14). Thus, although the nanotube surface Young’s modulus remains essentially independent of diameter and chirality within the investigated range, its relationship with the two directional moduli of the parent nanosheet varies across the group 13–phosphide compounds.

This correspondence should not, however, be interpreted as requiring that $E_{s}^{\mathrm{NT}}$ be between $E_{\mathrm{sx}}$ and $E_{\mathrm{sy}}$. The nanotube modulus is derived from the combined tensile and bending response of the tubular structure, while $E_{\mathrm{sx}}$ and $E_{\mathrm{sy}}$ characterize the uniaxial deformation of the planar nanosheet along two specific directions of the lattice. Representing the same hexagonal network in planar and tubular geometries alters how equivalent beam elements contribute to the overall mechanical response. The proximity of $E_{s}^{\mathrm{NT}}$ to $E_{\mathrm{sx}}$ or $E_{\mathrm{sy}}$ should therefore be considered a characteristic of the predicted mechanical response for each compound, and not a general geometrical requirement.

Finally, the influence of the input parameters on the surface Young’s modulus of the armchair (17,17) AlPNT was examined using the four sets of data listed in Table 5. The resulting $E_{s}^{\mathrm{NT}}$ values were 0.084, 0.086, 0.087, and 0.093 TPa·nm for the input parameters reported by Liu et al. [3], Singh et al. [37] (adopted in the present study), Faraji et al. [5], and Kourra et al. [6], respectively. The surface Young’s modulus values remain within a relatively narrow range, with a maximum relative difference of approximately 8.4%, showing that, similarly to the results obtained for AlP nanosheets, the predicted surface Young’s modulus is only moderately affected by the choice of input parameters.

After determining the surface Young’s moduli of the investigated nanotubes, the present results were compared with data obtained in the literature. However, the Young’s modulus values reported for group 13–phosphide nanotubes are scarce and do not cover the complete BP–AlP–GaP–InP–TlP series. Table 10 therefore summarizes the results available for one-dimensional nanostructures of BP, GaP, and InP. Due to the limited data available specifically for phosphide nanotubes, values reported for GaP and InP nanowires (NWs) were also included to extend the comparison to alternative one-dimensional forms of the corresponding compounds. In particular, no previous theoretical predictions of the elastic properties of TlP nanotubes were identified, making the present results an initial reference for this largely unexplored tubular structure. Their inclusion in the present study also enables a systematic comparison of the elastic response across the complete B–Al–Ga–In–Tl phosphide nanotube series.

The Young’s modulus, $E_{NT\left(W\right)}$, of InP nanowires (InPNWs) reported in the selected studies was determined using different approaches: MD simulations [62,63], a theoretical model based on the ultrasonic non-destructive evaluation technique [64], and experimental measurements based on bending using scanning probe microscopy (SPM) [65]. The Young’s modulus of GaP nanowires (GaPNWs) was also determined experimentally using an atomic force microscopy (AFM)-based bending-profile method [66].

To enable comparison based on a consistent surface-modulus, the Young’s modulus values reported in the literature as three-dimensional moduli were converted to surface Young’s moduli according to $E_{s}^{\mathrm{NT}\left(W\right)}\;=\;E_{\mathrm{NT}\left(W\right)}t_{n}$, using the corresponding effective thicknesses given in Table 10. Regarding phosphide nanotubes, the closest agreement is found for InPNTs, with a difference of approximately 7% between the value of $E_{s}^{\mathrm{NT}}$ in the current study and that reported by Jiang and Guo [31]. These authors employed a stick-and-spring analytical model within the NCM/MSM framework, providing a modeling approach conceptually close to that adopted in the present study. In contrast, a considerably larger discrepancy is observed for BPNTs. Rocha et al. [29], using DFT calculations with the GGA-PBE functional to describe exchange–correlation interactions, as implemented in VASP, reported substantially higher Young’s moduli for BPNTs. After conversion to surface Young’s modulus using the adopted effective thickness, their values correspond to 0.472 and 0.305 TPa·nm for armchair and zigzag BPNTs, respectively, markedly exceeding both the present value of 0.175 TPa·nm and the 0.118 TPa·nm reported by Jiang and Guo [31].

**Table 10:** Comparison of the current results for the surface Young’s modulus of BP, GaP, and InP nanotubes with values reported in the literature for the corresponding nanotubes and nanowires.

Reference	NT/NW	$E_{s}^{\mathrm{NT}(W)}$, TPa·nm	Comments
Jiang and Guo [31]	BPNTs	0.118	Average value estimated from converged $\left(n,\;n\right)$ and $\left(n,\;0\right)$ NT results
	InPNTs	0.051	
Rocha et al. [29]	BPNTs	0.472 *	Average value for (*n*, *n*) NTs
		0.305 *	average value for (*n*, 0) NTs
Islam et al. [63]	InPNWs	0.046 *	ZB [111] NW, cross-section of 3.594 × 3.734 nm^2^
Pial et al. [62]		0.050 *	ZB NW, cross-sectional width ranging from 2.93 to 5.87 nm **
		0.040 *	WZ NW, cross-sectional width ranging from 2.49 to 6.21 nm **
Tripathi et al. [64]		0.053 *	WZ NW, diameter ranging from 0.81 to 5 nm
Dunaevskiy et al. [65]		0.056 *	Conical WZ NW, average diameter ranging from 34 to 80 nm
Alekseev et al. [66]	GaPNWs	0.064 *	Conical WZ NW, average diameter ranging from 60 to 130 nm
Current values	BPNTs	0.175	Analytical value obtained from Equation (25)
	GaPNTs	0.077	
	InPNTs	0.055	

* Calculated as $E_{s}^{\mathrm{NT}(W)}\;={\;E}_{\mathrm{NT}(W)}\;t_{n}$, using $t_{n}\;=\;0.372,\;0.415$ and 0.429 nm for BP, GaP and InP 1D nanostructures, respectively. ** Cross-sectional height-to-width ratio, H/D = 1 (ZB) and 1.155 (WZ).

The present $E_{s}^{\mathrm{NT}}$ value for InPNTs shows good agreement with the converted surface Young’s modulus values obtained from the InPNW results reported by Dunaevskiy et al. [65] and Tripathi et al. [64], with differences of only 2.2% and 2.5%, respectively. Pial et al. [62] investigated both WZ and ZB InPNWs and found a considerable influence of the crystal structure on their Young’s modulus. The corresponding converted $E_{s}^{\mathrm{NW}}$ values for the WZ and ZB structures are approximately 9% and 35% lower, respectively, than the present $E_{s}^{\mathrm{NT}}$ value for InPNTs. On the other hand, Pial et al. [62] found that the Young’s modulus remains almost constant with increasing NW size, a behavior comparable to the weak diameter dependence observed for the phosphide nanotubes in the present study.

The converted $E_{s}^{\mathrm{NW}}$ value obtained from the InPNW results of Islam et al. [63] is approximately 19% lower than the present value for InPNTs. A similar difference is observed for GaP, with the converted surface Young’s modulus obtained from the GaPNW measurements of Alekseev et al. [66] being approximately 19% lower than the present value for GaPNTs. Alekseev et al. [66] also investigated both WZ and ZB GaPNWs but, in contrast to the results reported by Pial et al. [62] for InPNWs, found the influence of crystal structure on the Young’s modulus to be negligible.

Overall, despite the structural differences between hollow nanotubes with a hexagonal diatomic arrangement and solid nanowires of the same binary compounds, their surface Young’s moduli are of comparable magnitude. For InP, some of the available NW results are very close to the present InPNT value, whereas others, as well as the available GaPNW result, are moderately lower. The magnitude of these differences depends on the compound, crystal structure, and study considered. These findings indicate that the surface Young’s moduli values of the investigated phosphide nanotubes are broadly comparable in magnitude to those reported for the corresponding nanowire results, although the differences depend on the compound, crystal structure, and study considered.

Following the analysis of the surface Young’s modulus, the shear response of the group 13–phosphide nanotubes was examined in terms of the surface shear modulus, $G_{s}^{\mathrm{NT}}$. According to Equation (17), $G_{s}^{\mathrm{NT}}$ is determined from the tensile, bending, and torsional rigidities obtained from the FE simulations. As established in Section 3.2.1, these rigidities are accurately represented by Equations (22)–(24) using the corresponding fitting parameters. Their substitution into Equation (17) eliminates the explicit dependence on nanotube diameter and yields

(26) $$G_{s}^{\mathrm{NT}}=\frac{\gamma_{\mathrm{A13–P}}}{\pi \sqrt{32{\left(\frac{{\;\beta }_{A13-P}}{{\;\alpha }_{A13-P}}\right)}^{3}}},$$

where $\alpha_{\mathrm{A13–P}}$, $\beta_{\mathrm{A13–P}}$ and $\gamma_{\mathrm{A13–P}}$ are the fitting parameters associated with the tensile, bending, and torsional rigidities, respectively, as reported in Table 9.

**Figure 15:**
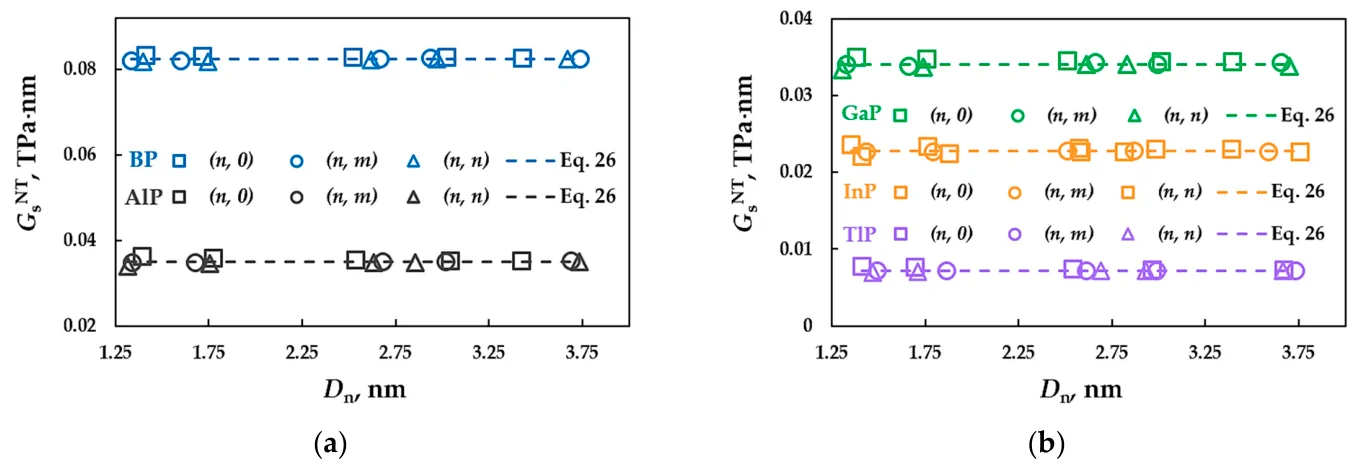
Surface shear modulus, $G_{s}^{\mathrm{NT}}$, as a function of nanotube diameter, $D_{n}$, for (**a**) SWBPNTs and SWAlPNTs and (**b**) SWGaPNTs, SWInPNTs, and SWTlPNTs. The corresponding analytical values predicted by Equation (26) are also shown.

The surface shear moduli, $G_{s}^{\mathrm{NT}}$, calculated from the FE rigidities using Equation (17) are presented in Figure 15a,b, together with the analytical estimates obtained from Equation (26). Overall, their values exhibit limited variation over the investigated diameter range, for each group 13–phosphide compound. The analytical values given by Equation (26) closely represent the FE results, with average relative differences of approximately 0.4%, 0.9%, 1.0%, 1.1%, and 2.0% for BPNTs, AlPNTs, GaPNTs, InPNTs, and TlPNTs, respectively. Equation (26) therefore provides a suitable analytical estimate of the surface shear modulus for the investigated nanotubes with $D_{n}>1.3$ nm.

**Figure 16:**
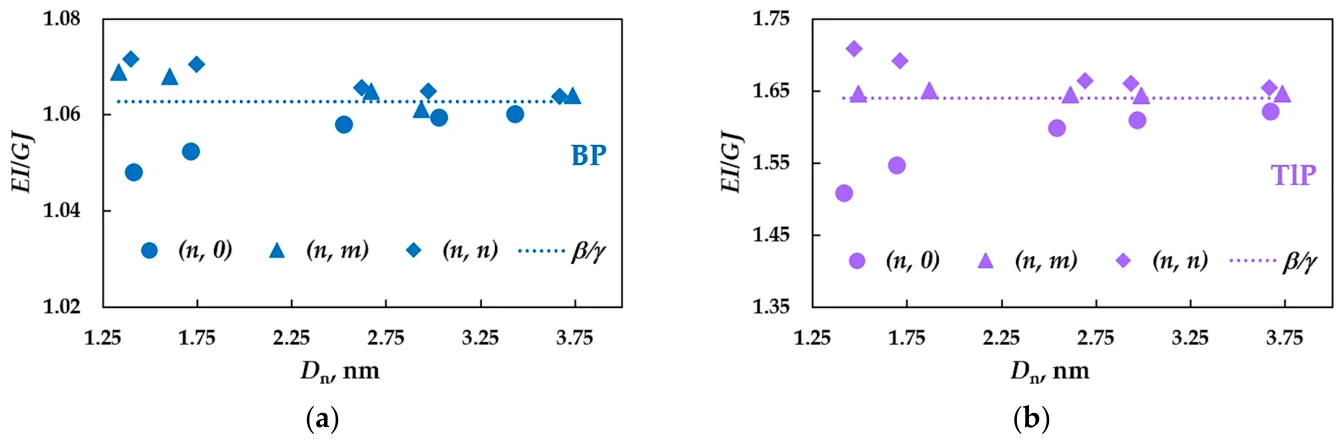
Ratio between bending and torsional rigidities, EI/GJ, as a function of nanotube diameter, $D_{n}$, for (**a**) SWBPNTs and (**b**) SWTlPNTs. The corresponding $\beta_{\mathrm{A13–P}}/\gamma_{\mathrm{A13–P}}$ ratios obtained from Equations (23) and (24) are also shown.

Unlike the surface Young’s modulus, the agreement between the $G_{s}^{\mathrm{NT}}$ values obtained directly from the FE rigidities and those predicted by Equation (26) gradually decreases along the B–Al–Ga–In–Tl series, although the average relative difference remains small, reaching approximately 2.0% for TlPNTs. To gain further insight into this behavior, we examined the ratio between the bending and torsional rigidities, EI/GJ, which enters directly into the evaluation of the surface shear modulus through Equation (17). Figure 16 illustrates the evolution of the ratio EI/GJ with nanotube diameter, for the two limiting compounds of the series, BP and TlP.

For both compounds, the EI/GJ ratio exhibits a discernible dependence on nanotube diameter and chirality at the lower end of the investigated diameter range. In general, EI/GJ increases with $D_{n}$ for zigzag $\left(n,\;0\right)$ nanotubes, while a decreasing or much weaker variation is observed for chiral $\left(n,\;m\right)$ and armchair $\left(n,\;n\right)$ configurations. As the diameter increases, the values obtained for the different chiralities approach the corresponding $\beta_{\mathrm{A13–P}}/\gamma_{\mathrm{A13–P}}$ ratio derived from Equations (23) and (24). This convergence is faster for BPNTs, which also exhibit a narrower spread of the EI/GJ values. In contrast, TlPNTs retain larger variations among the individual configurations over a wider diameter range. This behavior is consistent with the somewhat larger discrepancy between the surface shear modulus predicted by Equation (26) and that calculated directly from the FE rigidities for TlPNTs.

**Figure 17:**
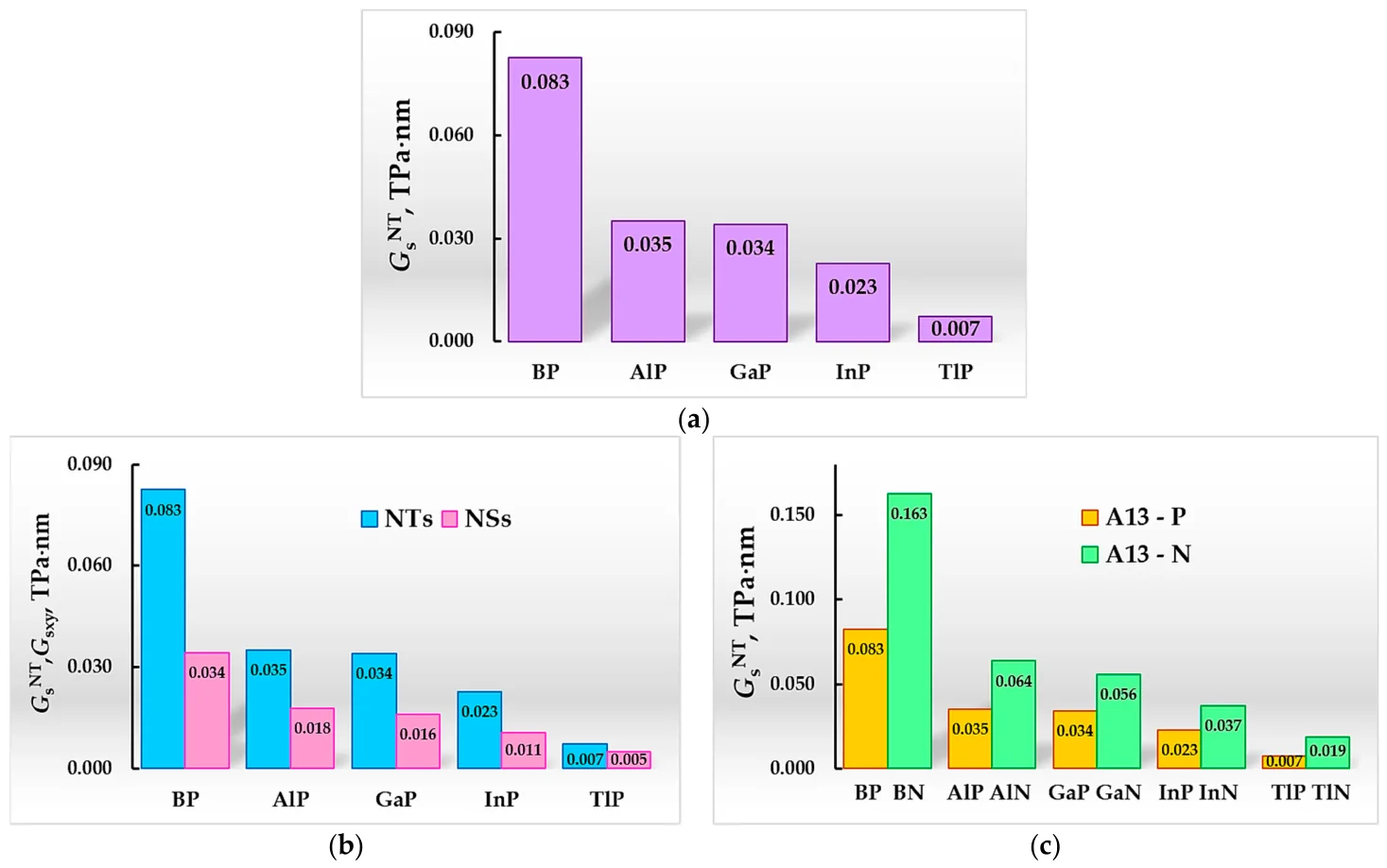
(**a**) Surface shear modulus, $G_{s}^{\mathrm{NT}}$, of group 13–phosphide nanotubes; comparison with (**b**) the surface shear modulus, $G_{\mathrm{sxy}}$, of the corresponding group 13–phosphide nanosheets and (**c**) $G_{s}^{\mathrm{NT}}$ of the corresponding group 13–nitride nanotubes [42,61].

The surface shear modulus, $G_{s}^{\mathrm{NT}}$, predicted by Equation (26) is presented for the B–Al–Ga–In–Tl series in Figure 17a. A pronounced reduction is observed from BPNTs to AlPNTs, with $G_{s}^{\mathrm{NT}}$ decreasing by a factor of approximately 2.4. The values for AlPNTs and GaPNTs are very similar, while a further decrease of approximately 1.5 times occurs from GaPNTs to InPNTs. The largest relative reduction is observed between InPNTs and TlPNTs, for which $G_{s}^{\mathrm{NT}}$ decreases by a factor of approximately 3.3. Overall, the surface shear modulus shows a marked, although non-uniform, decrease along the group 13–phosphide series.

The sensitivity of $G_{s}^{\mathrm{NT}}$ to the input parameters was further assessed for the armchair (17,17) AlPNT using the four datasets listed in Table 5. Depending on the adopted dataset, $G_{s}^{\mathrm{NT}}$ ranged from 0.035 to 0.042 TPa·nm, with values of 0.035, 0.036, 0.038, and 0.042 TPa·nm obtained using the parameters reported by Singh et al. [37], Kourra et al. [6], Liu et al. [3], and Faraji et al. [5], respectively. The first three datasets yield closely comparable predictions, differing by no more than approximately 8.8%, whereas the parameters reported by Faraji et al. [5] result in a value approximately 21% higher than that obtained with the Singh et al. [37] dataset, adopted in the present study. Thus, although the predicted $G_{s}^{\mathrm{NT}}$ is relatively consistent for most of the datasets considered, its magnitude can be appreciably affected by the specific set of force field parameters employed. This sensitivity should be taken into account when interpreting relatively small differences in $G_{s}^{\mathrm{NT}}$, such as those observed between AlPNTs and GaPNTs.

To place the nanotube results in the context of the corresponding 2D nanostructures, Figure 17b compares $G_{s}^{\mathrm{NT}}$ with the surface shear modulus, $G_{\mathrm{sxy}}$, obtained for the respective nanosheets. Unlike the surface Young’s moduli discussed above, the shear moduli of NTs and NSs are associated with different deformation modes—torsion of the nanotubes and in-plane shear of the nanosheets, respectively—and therefore should not be interpreted as directly equivalent quantities. Nevertheless, the comparison provides an indication of their relative resistance to shear deformation within the present modeling framework. The $G_{s}^{\mathrm{NT}}$ values are approximately 2.4, 2.0, 2.1, 2.1, and 1.5 times higher than the corresponding $G_{\mathrm{sxy}}$ values for BP, AlP, GaP, InP, and TlP, respectively. Thus, despite the different deformation modes involved, the investigated phosphide nanotubes consistently exhibit higher surface shear moduli than their nanosheet counterparts. Importantly, this difference should not be attributed solely to the tubular geometry, since the different deformation modes must also be considered.

To complete the analysis, the surface shear moduli of the group 13–phosphide nanotubes were compared with those previously obtained for the corresponding group 13–nitride nanotubes [42,61], as shown in Figure 17c. The $G_{s}^{\mathrm{NT}}$ values of BPNTs, AlPNTs, GaPNTs, InPNTs, and TlPNTs are approximately 50%, 45%, 40%, 40%, and 60% lower, respectively, than those of the corresponding nitride nanotubes. Moreover, the decrease in $G_{s}^{\mathrm{NT}}$ along the nitride series follows a comparatively gradual trend, whereas a less uniform evolution is observed for the phosphide nanotubes, with more pronounced reductions from BPNTs to AlPNTs and from InPNTs to TlPNTs.

### 3.3. Outlook and Future Challenges

The present study provides a systematic description of the elastic behavior of group 13–phosphide nanosheets and nanotubes across the B–Al–Ga–In–Tl series within a common NCM/MSM framework. Nevertheless, the scarcity of available theoretical data and, in particular, experimental data remains a major challenge in assessing the mechanical response of these nanostructures. Further modeling efforts employing complementary computational approaches, together with experimental investigations, would therefore be valuable for validating the predicted elastic properties and trends and for establishing more comprehensive reference data for group 13–phosphide nanostructures. The systematic set of results generated in the present study may also provide useful reference data for future comparative studies, and contribute to the development and validation of data-driven models for predicting the mechanical of related nanostructures.

The NCM/MSM approach adopted here provides an efficient framework for investigating relatively large nanostructures while retaining the discrete character of the atomic network through equivalent beam elements. It also enables the systematic analysis of nanostructures with different geometries and dimensions at comparatively low computational cost. However, the model does not explicitly account for electronic structure, bond rearrangement, defect formation, or other atomistic mechanisms that may become important under large deformation or in the vicinity of failure. The investigation of nonlinear deformation, strength, and failure mechanisms would therefore require complementary modeling approaches capable of capturing these effects.

Further investigation is also required to better understand the non-uniform trends observed across the phosphide series, particularly the distinctive behavior of TlP-based nanostructures. As demonstrated by the sensitivity analyses performed for AlP, the predicted elastic properties depend on the adopted force field parameters. For TlP, the limited availability of published material properties restricts the determination of alternative sets of force field parameters, and the sensitivity of the present predictions to different input data remains to be assessed. Complementary computational studies could provide deeper insight into the relationship between the force field parameters adopted in the NCM/MSM formulation and the resulting overall elastic response. They could also help to elucidate the similarities and differences identified in the mechanical behavior of corresponding nanosheets and nanotubes. Such investigations would extend the present continuum–molecular structural analysis and contribute to a more comprehensive description of the mechanical behavior of group 13–phosphide nanostructures.

## 4. Conclusions

This study provides a systematic investigation of the elastic behavior of group 13–phosphide nanosheets and single-walled nanotubes across the B–Al–Ga–In–Tl series using a common NCM/MSM framework couple with finite element analysis. By considering the 2D and 1D forms within the same modeling approach, their mechanical responses can be consistently compared, allowing a systematic assessment of the evolution of the elastic properties across the group 13–phosphide series for both types of nanostructures. This is particularly relevant given the limited availability of mechanical data for group 13–phosphide nanostructures, especially nanotubes, for which no previous elastic data for TlPNTs were identified.

The results reveal an overall decrease in elastic properties along the B–Al–Ga–In–Tl series, although the evolution is not uniform across all compounds and properties. For the nanosheets, the average surface Young’s modulus decreases from 0.175 TPa·nm for BP to 0.025 TPa·nm for TlP, while the surface shear modulus decreases from 0.034 to 0.005 TPa·nm. For the corresponding nanotube, the surface Young’s moduli are approximately 0.175, 0.086, 0.078, 0.055, and 0.024 TPa·nm for BP, AlP, GaP, InP, and TlP, respectively. These values exhibit only a weak dependence on nanotube diameter and chirality within the investigated range ($D_{n}>1.3$ nm). In contrast, the surface shear moduli follow a less regular trend, with corresponding values of approximately 0.083, 0.035, 0.034, 0.023, and 0.007 TPa·nm, respectively.

The combined analysis of the 2D and 1D structures reveals that the surface Young’s moduli of the nanotubes are close to those of the corresponding nanosheets, although their correspondence with the zigzag and armchair nanosheet directions varies across the phosphide series. The comparison of the shear response shows consistently higher surface shear moduli for the nanotubes than for the corresponding nanosheets. However, these values arise from different deformation modes—torsion of the nanotubes and in-plane shear of the nanosheets—and should therefore not be regarded as directly equivalent measures of shear resistance. Overall, these results provide a consistent basis for comparing the elastic response of the 2D and 1D forms of the investigated phosphides, highlighting both the common trends and compound-dependent differences between them.

For the group 13–phosphide nanotubes, the established relationships between tensile, bending, and torsional rigidities and nanotube diameter were confirmed across the B–Al–Ga–In–Tl series. The newly determined fitting parameters $\alpha_{\mathrm{A13–P}}$, $\beta_{\mathrm{A13–P}}$, and $\gamma_{\mathrm{A13–P}}$ provide an accurate analytical estimation of the corresponding surface Young’s and shear moduli, with only small average relative differences from the values calculated directly from the FE rigidities. The sensitivity analysis performed for AlP further showed that the predicted elastic moduli are reasonably consistent across most of the input datasets considered. It is also highlighted the importance of the selection of force field parameters when interpreting relatively small differences in elastic properties between compounds.

Overall, the present study establishes new elastic properties and rigidity-related parameters for group 13–phosphide nanostructures across the complete B–Al–Ga–In–Tl series. These results provide a useful reference for future computational and experimental studies and support broader comparative investigations of the mechanical behavior of related 2D and 1D materials.

## Figures and Tables

**Table 1:** Dimensions of the BP, AlP, GaP, InP and TlP nanosheets considered in this study.

Compound	NS Size ^1^, $L_{x}\times {\;L}_{y},$ nm^2^
BP	15.21 × 15.74
AlP	16.73 × 15.64
GaP	15.80 × 15.48
InP	15.34 × 16.03
TlP	14.89 × 15.91

^1^ Dimensions of approximately 15 × 15 nm^2^ were selected, for which the mechanical response of the NSs no longer depends on their size [34].

**Table 2:** Chiral indices and diameters of the single-walled zigzag, chiral and armchair BP, AlP, GaP, InP and TlP nanotubes.

NT	Zigzag (*n*, 0), *θ* = 0°			Chiral (*n*, *m*), *θ* = 19.1°			Armchair (*n*, *n*), *θ* = 30°		
	(*n*, *m*)	*D*_n_, nm	*L*_z_, nm **	(*n*, *m*)	*D*_n_, nm	*L*_z_, nm **	(*n*, *m*)	*D*_n_, nm	*L*_z_, nm **
SWBPNTs	(14, 0)	1.413	41.82	(10, 5)	1.335	39.84	(8, 8)	1.398	42.00
	(17, 0)	1.715	51.70	(12, 6)	1.602	48.88	(10, 10)	1.748	53.70
	(25, 0)	2.522	76.40	(20, 10)	2.669	80.83	(15, 15)	2.621	78.42
	(30, 0)	3.027	91.23	(22, 11)	2.936	87.95	(17, 17)	2.971	89.82
	(34, 0)	3.430	102.75	(28, 14)	3.737	112.77	(19, 19)	3.320	99.33
SWAlPTs *	(11, 0)	1.395	41.52	(8, 4)	1.342	42.55	(6, 6)	1.318	39.84
	(14, 0)	1.775	53.94	(10, 5)	1.677	51.67	(8, 8)	1.757	54.18
	(20, 0)	2.536	76.71	(16, 8)	2.684	80.69	(12, 12)	2.636	80.08
	(24, 0)	3.043	91.20	(18, 9)	3.019	91.82	(13, 13)	2.855	86.05
	(27, 0)	3.424	103.62	(22, 11)	3.690	111.72	(17, 17)	3.734	113.94
SWGaPNTs *	(11, 0)	1.383	41.50	(8, 4)	1.330	39.60	(6, 6)	1.306	39.49
	(14, 0)	1.760	54.15	(10, 5)	1.663	51.24	(8, 8)	1.742	52.93
	(20, 0)	2.514	76.04	(16, 8)	2.661	80.03	(12, 12)	2.613	79.79
	(24, 0)	3.017	91.09	(18, 9)	2.993	91.06	(13, 13)	2.830	86.11
	(27, 0)	3.394	102.03	(22, 11)	3.658	108.99	(17, 17)	3.701	111.89
SWInPNTs	(10, 0)	1.356	39.61	(8, 4)	1.435	42.72	(6, 6)	1.409	42.81
	(13, 0)	1.763	52.12	(10, 5)	1.794	55.08	(8, 8)	1.879	57.72
	(19, 0)	2.577	78.67	(14, 7)	2.512	76.55	(11, 11)	2.584	79.02
	(22, 0)	2.984	90.53	(16, 8)	2.871	86.07	(12, 12)	2.819	85.41
	(25, 0)	3.391	102.27	(20, 10)	3.588	107.83	(16, 16)	3.759	113.10
SWTlPNTs	(10, 0)	1.412	43.52	(8, 4)	1.494	44.90	(6, 6)	1.467	44.58
	(12, 0)	1.694	51.20	(10, 5)	1.868	57.09	(7, 7)	1.712	51.24
	(18, 0)	2.542	77.31	(14, 7)	2.615	79.49	(11, 11)	2.690	82.29
	(21, 0)	2.965	89.60	(16, 8)	2.989	89.60	(12, 12)	2.935	88.94
	(24, 0)	3.671	111.11	(20, 10)	3.736	112.05	(15, 15)	3.668	111.12

* The diameters, $D_{n}$, of SWAlPNTs and SWGaPNTs were calculated using AlP and GaP equilibrium bond lengths of 0.230 nm and 0.228 nm, respectively, as defined in Nanotube Modeler© software. ** The NT length, $L_{z}$, was set to approximately 30 times the nanotube diameter to ensure that the mechanical response no longer depends on the NT length [35].

**Table 3:** Material parameters and force field constants used in the modeling of group 13–phosphide nanostructures.

Compound	$a_{\mathrm{A13–P}}$, nm [1]	*E*_s_, nN/nm [1]	ν [1]	$k_{r}$, nN/nm	$k_{\theta }$, nN·nm/rad^2^	$k_{\tau }$, nN·nm/rad^2^
BP	0.183	135	0.28	325	0.709	0.625
AlP	0.23562 ^1^	57.3 ^1^	0.47 ^1^	188	0.380	
GaP	0.225	59	0.35	157	0.421	
InP	0.246	39	0.43	119	0.298	
TlP	0.2561 ^2^	13.6 ^2^	0.795 ^2^	115	0.076	

^1^ Values from Singh et al. [37]; ^2^ Values from Liu et al. [3].

**Table 4:** Geometrical and elastic properties of the equivalent beam elements used in the finite element simulations.

Compound	Diameter, *d*, nm	Formulation	Young’s Modulus, *E*_b_, GPa	Formulation	Shear Modulus, *G*_b_, GPa	Formulation	Poisson’s Ratio, $\nu_{b\;}$ [S]
BP	0.1869	$\;d=4\sqrt{\frac{k_{\theta }}{k_{r}}}$	2165	$E_{b}=\frac{k_{r}^{2}l}{4\pi k_{\theta }}$	954	$G_{b}=\frac{{k_{r}^{2}k}_{\tau }l}{8\pi \;k_{\theta }^{2}}$	0.28
AlP	0.1800		1740		1430		0.47 ^1^
GaP	0.2069		1052		782		0.35
InP	0.2004		924		971		0.43
TlP	0.1029		3538		14,532		0.795 ^2^

^1^ Values from Singh et al. [37]; ^2^ values from Liu et al. [3].

**Table 5:** Material parameters ($a_{\mathrm{Al}-P}$, *E*_s,_ ν), force field constants ($k_{r}$, $k_{\theta }$), and geometrical (*d*) and elastic (*E*_b_, *G*_b_,) properties of equivalent beams derived from different input data sets for AlP nanostructures.

Reference	$a_{Al-P}=l$, nm	*E*_s_, nN/nm	$\nu =\nu_{b\;}$	$k_{r}$, nN/nm	$k_{\theta }$, nN·nm/rad^2^	*d*, nm	*E*_b_, GPa	*G*_b_, GPa
Singh et al. [37] ^1^	0.23562	57.3	0.47	188	0.380	0.1800	1740	1430
Liu et al. [3]	0.2277	64.4	0.313	162	0.497	0.2213	0.1107	960
Faraji et al. [5]	0.234	66.3	0.25	153	0.599	0.2502	729	380
Kourra et al. [6]	0.227	65.6	0.5	227	0.390	0.1658	2390	1913

^1^ Adopted in the current study.

**Table 6:** Surface Young’s moduli, $E_{\mathrm{sx}}$ and $E_{\mathrm{sy}}$, and their ratio, $E_{\mathrm{sx}}/E_{\mathrm{sy}}$, obtained for the AlPNS using the different sets of input parameters listed in Table 5.

Reference	$E_{\mathrm{sx}}$, TPa·nm	$E_{\mathrm{sy}}$, TPa·nm	$E_{\mathrm{sx}}/E_{\mathrm{sy}}$
Singh et al. [37] ^1^	0.088	0.086	1.016
Liu et al. [3]	0.085	0.084	1.016
Faraji et al. [5]	0.088	0.087	1.015
Kourra et al. [6]	0.096	0.094	1.016

^1^ Adopted in the current study.

**Table 7:** Comparison of the present elastic moduli with literature values for group 13–phosphide NSs.

Year	Reference	Compound	$E_{\mathrm{sx}}$, TPa⋅nm	$E_{\mathrm{sy}}$, TPa⋅nm	$E_{\mathrm{sx}}/E_{\mathrm{sy}}$	$E_{s}^{\mathrm{NS}}$, TPa⋅nm	$G_{\mathrm{sxy}}$, TPa⋅nm
2012	Zhuang and Hennig [43]	BP				0.172	
2019	Yuan et al. [13]	TlP				0.022	
	Liu et al. [3]	BP					0.054
		AlP					0.020
		GaP					0.021
		InP					0.014
		TlP					0.011
2020	Mohanta et al. [18]	BP	0.152	0.153	0.998	0.153	0.065
2021	Singh et al. [37] ^1^	BP	0.144	0.139	1.033	0.141	0.053
		AlP					0.019
	Vu et al. [15]	BP				0.136	0.054
2022	Mondal et al. [10]					0.133	0.052
	Galicia-Hernandez et al. [12]					0.101 ^2^	0.038 ^3^
2025	Gajjar et al. [11]		0.1367	0.1371	0.997	0.137	0.054
		GaP	0.0553	0.0554	0.998	0.055	0.021
		InP	0.0375	0.0377	0.995	0.038	0.013
2026	Current study	BP	0.176	0.174	1.011	0.175	0.034
		AlP	0.088	0.086	1.016	0.087	0.018
		GaP	0.079	0.078	1.019	0.078	0.016
		InP	0.056	0.054	1.035	0.055	0.011
		TlP	0.027	0.023	1.155	0.025	0.005

^1^ Nanosheet dimensions: 10.3891 × 12.2105 nm^2^ for BP and 13.1946 × 15.5078 nm^2^ for AlP. ^2^ Calculated from the reported elastic constants [12] using $E_{s}^{\mathrm{NS}}\;=\;\left(C_{11}^{2}\;-{\;C}_{12}^{2}\right)/C_{11}$. ^3^ Calculated from the reported elastic constants [12] using $G_{\mathrm{sxy}}=\left(C_{11}\;-\;C_{12}\right)/2$.

**Table 8:** Comparison of the Young’s and shear moduli obtained for the 2D group 13–phosphide monolayers in the present study with literature values for the corresponding 3D compounds.

Reference	Compound	$E_{\mathrm{A13–P}},$ TPa		$G_{\mathrm{A13–P}}$, TPa	
		Ref.	Current	Ref.	Current
Abdallah et al. [28]	BP	0.241 ^1^	0.469	0.096 ^2^	0.092
Xia et al. [44]		0.351		0.158	
Mahat et al. [45]		0.335 ^1^		0.136 ^2^	
Ren et al. [56]		0.406		0.143 ^2^	
Daoud [46]	AlP	0.121	0.239	0.048	0.049
Yang et al. [47]		0.119		0.047	
Bouhemadou et al. [48]		0.113		0.044	
Fayyadh [49]		0.124		0.048	
Elkenany et al. [52]		0.097		0.037	
Fayyadh [50]	GaP	0.142	0.189	0.056	0.038
Ribeiro-Silva et al. [57]		0.102		0.039	
Wang et al. [60]		0.103		0.039	
Hajlaoui et al. [53]	InP	0.094 ^1^	0.128	0.033	0.025
Pruessner et al. [58]		0.105			
Miao et al. [55]	AlP	0.132	0.239	0.053	0.049
	GaP	0.140	0.189	0.058	0.038
	InP	0.094	0.128	0.037	0.025
Ehsanfar et al. [51]	BP	0.317	0.469	0.135	0.092
	AlP	0.115	0.239	0.045	0.049
	GaP	0.126	0.189	0.050	0.038
	InP	0.099	0.128	0.041	0.025
Tripathi et al. [9]	BP	0.272	0.469	0.112	0.092
	AlP	0.138	0.239	0.057	0.049
	GaP	0.151	0.189	0.062	0.038
	InP	0.091	0.128	0.035	0.025
Wang and Ye [54]	BP	0.329	0.469	0.173 ^2^	0.092
	AlP	0.088	0.239	0.048 ^2^	0.049
	GaP	0.114	0.189	0.061 ^2^	0.038
	InP	0.0719	0.128	0.040 ^2^	0.025
	TlP	0.0724	0.057	0.041 ^2^	0.011

^1^ Calculated from the reported elastic constants [28,45,53] using $E=\;(C_{11}^{2}-C_{12}^{2})$. ^2^ Calculated from the reported elastic constants [28,45,54,56] using $G\;=\;(C_{11}-C_{12})/2$.

**Table 9:** Fitting parameters $\alpha_{\mathrm{A13–P}}$, $\beta_{\mathrm{A13–P}}$ and $\gamma_{\mathrm{A13–P}}$, and mean relative differences between the rigidities predicted by Equations (22)–(24) and the corresponding FE results obtained from Equations (13)–(15).

Compound	Fitting Parameters			Mean Difference, %		
	${\;\alpha }_{\mathrm{A13–P}}$, nN/nm	${\;\beta }_{\mathrm{A13–P}}$, nN/nm	$\gamma_{\mathrm{A13–P}}$, nN/nm	*EA*, nN	*EI*, nN·nm^2^	*GJ*, nN·nm^2^
BP	551.01 ^1^	68.82 ^1^	64.75 ^1^	0.15	0.41	0.11
AlP	269.43	33.61	27.44	0.36	0.83	0.46
GaP	243.21 ^1^	30.45 ^1^	26.83 ^1^	0.31	0.84	0.38
InP	171.35 ^1^	21.40 ^1^	17.84 ^1^	0.37	0.89	0.46
TlP	74.22	9.25	5.64	0.39	0.83	0.51

^1^ The fitting parameters obtained for BPNTs, GaPNTs, and InPNTs are close to those reported in our previous study [33].

## Data Availability

Additional data supporting the findings of this study are provided in the Supplementary Materials. Further data are available from the corresponding author upon reasonable request, subject to the necessary authorizations.

## Supplementary Materials

The following supporting information can be downloaded at https://www.mdpi.com/article/10.3390/nano16211398/s1: Finite element implementation details; Figure S1: (a) Tensile rigidity, *EA*, (b) bending rigidity, *EI*, and (c) torsional rigidity, *GJ*, of group 13–phosphide nanotubes as functions of nanotube diameter, *D*_n_.

## Abbreviations

The following abbreviations are used in this manuscript:

2D	Two-dimensional
BP	Boron phosphide
AlP	Aluminum phosphide
GaP	Gallium phosphide
InP	Indium phosphide
TlP	Thallium phosphide
DFT	Density Functional Theory
NS	Nanosheet
1D	One-dimensional
NT	Nanotube
BPNT	Boron phosphide nanotube
AlPNT	Aluminum phosphide nanotube
GaPNT	Gallium phosphide nanotube
InPNT	Indium phosphide nanotube
TlPNT	Thallium phosphide nanotube
SWBPNT	Single-walled boron phosphide nanotube
SWAlPNT	Single-walled aluminum phosphide nanotube
SWGaPNT	Single-walled gallium phosphide nanotube
SWInPNT	Single-walled indium phosphide nanotube
SWTlPNT	Single-walled thallium phosphide nanotube
NCM	Nanoscale Continuum Modeling
MSM	Molecular Structural Mechanics
FE	Finite Element
MM	Molecular Mechanics
TB	Tersoff–Brenner
VASP	Vienna Ab initio Simulation Package
GGA	Generalized Gradient Approximation
PBE	Perdew–Burke–Ernzerhof
FEA	Finite Element Analysis
BPNS	Boron phosphide nanosheet
AlPNS	Aluminum phosphide nanosheet
GaPNS	Gallium phosphide nanosheet
InPNS	Indium phosphide nanosheet
TlPNS	Thallium phosphide nanosheet
BNNS	Boron nitride nanosheet
AlNNS	Aluminum nitride nanosheet
GaNNS	Gallium nitride nanosheet
InNNS	Indium nitride nanosheet
TlNNS	Thallium nitride nanosheet
MD	Molecular Dynamics
3D	Tree-dimensional
WZ	Wurtzite
ZB	Zinc blende
vdW	van der Waals
BNNT	Boron nitride nanotube
TlNNT	Thallium nitride nanotube
AlNNT	Aluminum nitride nanotube
NW	Nanowire
InPNW	Indium phosphide nanowire
GaPNW	Gallium phosphide nanowire
SPM	Scanning Probe Microscopy
AFM	Atomic Force Microscopy
