# Supplementary material for: Elastic Behavior of Group 13–Phosphide Nanosheets and Nanotubes: A Numerical Study
Source: Nanomaterials (Basel). 2026 Oct 31;16(21):1398. doi: 10.3390/nano16211398 (PMC13764970; doi:10.3390/nano16211398)
Supplement: Supplementary file 1 [file nanomaterials-16-01398-s001.zip › nanomaterials-4663520-supplementary.pdf]

Supplementary Materials

Elastic Behavior of Group 13–Phosphide Nanosheets and Nanotubes: A Numerical Study

Nataliya A. Sakharova [1,2,*], André F. G. Pereira [2] and Jorge M. Antunes [2,3]

Finite element implementation

The finite element (FE) analyses of the nanosheets and nanotubes were performed using ABAQUS 2020/Standard. Interatomic bonds were represented by two-node, three-dimensional Euler–Bernoulli beam elements (B33), with circular cross-sections and linear elastic material properties. A single Static, General analysis step was used, with NLGEOM disabled and the default increment and convergence tolerance settings.

At the fixed boundaries, all six degrees of freedom of the corresponding edge nodes were constrained. For the tensile tests of the nanosheets and nanotubes, the total applied axial force was uniformly distributed among the nodes at the loaded edge or end, respectively. In the nanosheet in-plane shear tests, the displacement in the x-direction was measured in the central region of the nanosheet. For the nanotube bending tests, the total transverse force was uniformly distributed among the nodes at the loaded end. For the nanotube torsion tests, the nodes at the loaded end were kinematically coupled to a reference point at which the torsional moment was applied, ensuring uniform transfer of the applied moment.

Under these linear elastic conditions, all analyses converged in a single increment. The numbers of nodes and elements varied with nanosheet and nanotube geometry, as the FE discretization followed the atomic structure directly, with nodes representing atoms and beam elements representing the corresponding interatomic bonds.

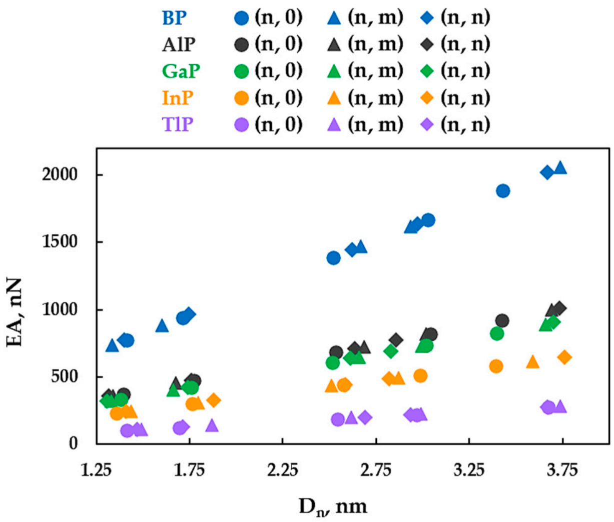

(a)

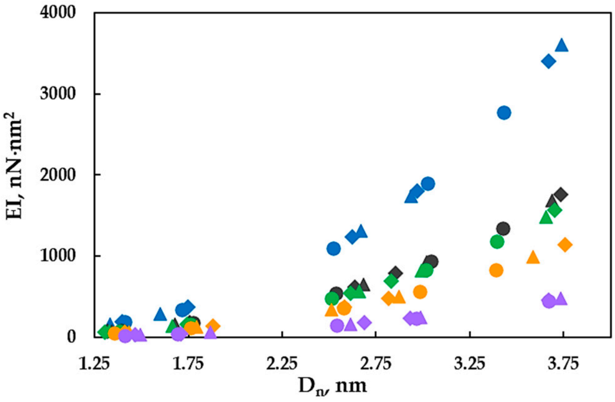

(b)

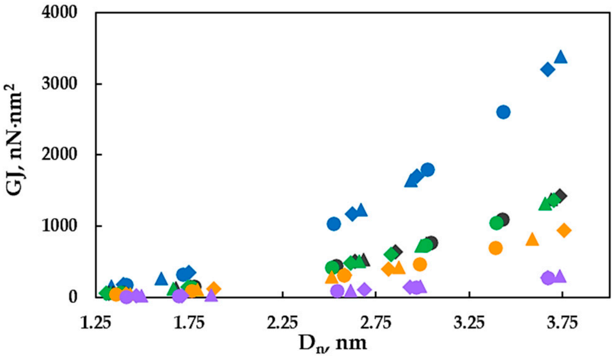

(c)

Figure S1. (**a**) Tensile rigidity, EA, (**b**) bending rigidity, EI, and (**c**) torsional rigidity, GJ, of group 13–phosphide nanotubes as functions of nanotube diameter, D_n.
